# A Particleboard Surface Defect Detection Method Research Based on the Deep Learning Algorithm

**DOI:** 10.3390/s22207733

**Published:** 2022-10-12

**Authors:** Ziyu Zhao, Zhedong Ge, Mengying Jia, Xiaoxia Yang, Ruicheng Ding, Yucheng Zhou

**Affiliations:** 1 School of Information and Electrical Engineering, Shandong Jianzhu University, Jinan 250101, China; 2 Research Institute of Wood Industry, Chinese Academy of Forestry, Beijing 100091, China

**Keywords:** surface defect detection, object detection, semantic segmentation, real-time, deep learning

## Abstract

Particleboard surface defects have a significant impact on product quality. A surface defect detection method is essential to enhancing the quality of particleboard because the conventional defect detection method has low accuracy and efficiency. This paper proposes a YOLO v5-Seg-Lab-4 (You Only Look Once v5 Segmentation-Lab-4) model based on deep learning. The model integrates object detection and semantic segmentation, which ensures real-time performance and improves the detection accuracy of the model. Firstly, YOLO v5s is used as the object detection network, and it is added into the SELayer module to improve the adaptability of the model to receptive field. Then, the Seg-Lab v3+ model is designed on the basis of DeepLab v3+. In this model, the object detection network is utilized as the backbone network of feature extraction, and the expansion rate of atrus convolution is reduced to the computational complexity of the model. The channel attention mechanism is added onto the feature fusion module, for the purpose of enhancing the feature characterization capabilities of the network algorithm as well as realizing the rapid and accurate detection of lightweight networks and small objects. Experimental results indicate that the proposed YOLO v5-Seg-Lab-4 model has mAP (Mean Average Precision) and mIoU (Mean Intersection over Union) of 93.20% and 76.63%, with a recognition efficiency of 56.02 fps. Finally, a case study of the Huizhou particleboard factory inspection is carried out to demonstrate the tiny detection accuracy and real-time performance of this proposed method, and the missed detection rate of surface defects of particleboard is less than 1.8%.

## 1. Introduction

Particleboard is a composite material made by blending wood, or other lignocellulosic materials. Both sides are made of fine wood fibers, which are formed by drying and hot pressing [1,2]. Defects appear on the surface of particleboard due to chemical composition, raw material ratios, technical processes, etc. Surface defects will seriously affect the stress strength of the plate, and cause difficulties in subsequent bonding. Up to now, the product classification of particleboard production lines still relies on manual experience to determine defects, and manual inspection has problems such as high labor intensity, low inspection efficiency and high false detection rate. Therefore, the development of an accurate and efficient real-time inspection system for particleboard surface defects is an effective measure to improve the quality of particleboard production [3,4].

Nondestructive inspection methods for wood panels include machine vision, ultrasonic, stress wave, and X-ray [5]. Zhang tested cedar wood using ultrasound and used two mathematical function models to regress the defect size on the ultrasound propagation velocity [6]. Only the quantitative relationship between ultrasonic propagation velocity and hole diameter was determined, but the defect types were not identified and classified. Wang utilized a defect detection classification method that combines stress wave technology with SVM to effectively distinguish defects such as insect eyes, knots and cracks, but it was difficult to distinguish between defective areas with less decay and normal areas of wood [7]. Qi also utilized X-ray detection of wood to obtain internal images and used artificial neural network algorithms to identify wood defects. However, the artificial neural network modeling process is complex, with many training parameters, and time-consuming [8].

The development of deep learning-based techniques has been accompanied by rapid advancements in computing power over the past few decades. Defect detection methods based on deep learning are divided into two main categories: object detection and semantic segmentation. An important application of computer vision is object detection, which finds and recognizes target objects in a scene and determines where the objects are located. For instance, Girshick proposed two-stage detectors, such as R-CNN (Region-CNN) [9]. R-CNN transforms the problem of object detection into a classification problem by employing CNN algorithms for the extraction of features and classification of objects. To alleviate the time-consuming problem caused by the RCNN, Faster R-CNN introduces RPN (Region Proposal Networks) that share convolutional layers with object detection networks. The marginal cost for computing proposals is small due to the sharing of convolutions during the test-time phase. [10] Furthermore, the one-stage methods mostly include YOLO [11,12,13,14], SSD (Single Shot MultiBox Detector) [15] and RetinaNet [16]. The YOLO method unifies object classification and localization into a regression problem; the detection speed has been greatly increased. YOLO has been successfully applied in the fields of medicine, traffic, remote sensing, agriculture, education and building construction. The YOLO series has been updated in five versions. A semantic segmentation task is defined as the problem of determining the semantic categorization between each pixel of an image. U-Net and DeepLab will be explored as the current representations of the best segmentation network for visible semantic segmentation [17,18]. The DeepLab model was first proposed by Google in 2015 for image semantic segmentation, based on fully convolutional networks. The DeepLab series has been updated four times since 2015 [19,20,21,22]. In the DeepLab series, a more successful ASPP structure was the output feature resolution can be accurately controlled by changing the atrous rates in order to effectively improve the segmentation accuracy.

Gradually, many researchers have investigated the detection of different surface defect objects on solid wood boards and rotary veneers using images [7,8,23,24,25,26]. However, there are still many open issues that remain challenging. Fan et al. used Faster R-CNN for the detection of defects in solid wood panels, and the correct rate of detecting wormhole defects in solid wood panels was 95%. However, the two-stage detection algorithm based on Faster R-CNN sacrifices the premise of real-time, thus improving the correctness of the algorithm, which does not meet the actual real-time demand [27]. Ye et al. utilized the LBP feature extraction algorithm to detect rotten knots, insect eyes and indentation defects on solid wood panels’ surfaces. The detection frame areas obtained were subject to misclassification and omission. The LBP algorithm was relatively single, and the overly tedious operations still led to poor real-time performance [28]. Some researchers have constructed a glance multi-channel mask regional convolutional neural network-integrated model, which includes glance network and multi-channel mask R-CNN, but the detection real-time performance needs to be enhanced [29]. Specifically, the surface of the particleboard is composed of fine particles, and the distribution of gray value in defects is completely different from that of the above boards. At the same time, after field research on the production site, it is concluded that the defect area only accounts for 0.2% of the total surface area of particleboard, and the difficulty lies in the simultaneous consideration of target detection at different scales, and the features of small-scale objects are difficult to identify. Furthermore, many existing studies have examined localization or proximity measurement techniques based on stereovision or marker-based methods. However, many previous detection models have been difficult to directly adopt in order to achieve high performance, balancing accuracy, and efficiency [30,31,32,33,34,35,36,37].

To address these issues, a real-time and multi-scale detection model based on particleboard surface defects is proposed. The first section is the proposal of a YOLO v5-Seg-Lab-4 model, which is the first employed for a fusion of object detection and semantic segmentation for particleboard surface defect detection. This enables the model to obtain more information on semantic features without compromising on real-time. The second section is the proposal of the RASPP (Reduced ASPP) and FCAM (Fusion Channel Attention Mechanism) modules, which effectively improve the feature characterization capabilities of the network. The model is able to identify information about small target objects more accurately and save computing time in order to achieve accurate identification of surface defects in particleboard. The model has been implemented in a particleboard factory in Huizhou. The application of the YOLO v5-Seg-Lab-4 model has solved the difficulties of small defect misjudgment and poor real-time inspection, resulting in increased economic advantages and labor savings for the company.

## 2. Model Construction

### 2.1. Algorithm Research Framework

**Figure 1:**
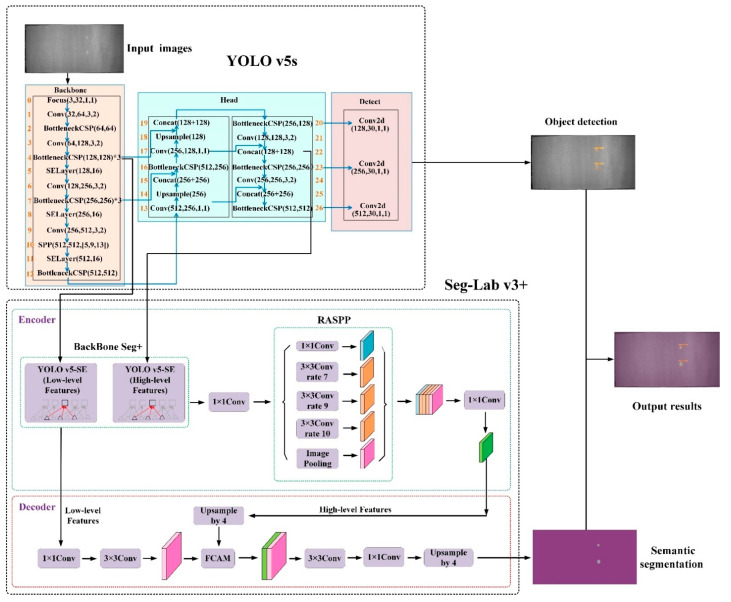
Research framework of YOLO v5-Seg-Lab-4.

Figure 1 depicts the research framework for a particleboard surface defect detection method that is both quick and accurate. To begin, YOLO v5s was utilized as the object detection network to detect surface flaws in particleboard quickly and at several scales. Then, to adapt to small target features, a Seg-Lab v3+ model based on DeepLab v3+ was provided, which simplified the model structure and parameters while maintaining model accuracy. The feature extraction backbone network was created with YOLO v5s, and RASPP and FCAM were used to increase the feature characterization capabilities of the network algorithm, making the algorithm more suitable for the detection of small target features.

### 2.2. YOLO v5s Network Architecture

The most lightweight version of the YOLO series and its network architecture is the YOLO v5s. The YOLO v5s is composed of three main parts in terms of Backbone, Head, and Detect. The Backbone is composed of three modules: the Focus module, the BottleneckCSP module, and the SPP (Spatial Pyramid Pooling) module [38,39,40]. The Focus module is designed to reduce the number of layers to increase speed. The BottleneckCSP module is modeled on the concepts of the cross-local network connection platform CSPNet in 2019 to reduce model parameters from the perspective of network construction, thereby improving reasoning speed while maintaining model accuracy. The SPP module is feature extraction and then the aggregation of a feature map from different angles to improve the robustness of the algorithm. In the Head section, the PAN structure is employed not only as a feature intensifier, but also to move the enhanced features from bottom to top to the prediction layer, improving the potential for network feature aggregation. The Detect module is used to predict feature map objects by different scales.

The form of surface defects in the feature map gradually blurs as the number of network layers increases, which can easily lead to errors. Therefore, this paper incorporates the SELayer attention mechanism into the backbone of YOLO v5s, added after BottleneckCSP and SPP. The SELayer attention mechanism works well as a plug-and-play module that integrates well with other model structures. First, the feature map of H × W × C is compressed into 1 × 1 × C, and the importance of each channel is predicted using the fully connected layer to obtain the importance magnitude of different channels. Then, a weighting operation is performed to multiply the learned activation values of each channel by the corresponding channel of the previous feature map, with a sigmoid activation value interval of 0 to 1, to obtain the output module output results, increasing the sensitivity of the model to channel and spatial features without reducing the speed of model detection. It effectively reduces the complexity of network feature learning and fits the data better, thus increasing the adaptive capability of the model to the perceptual field.

### 2.3. Seg-Lab v3+ Algorithm

The Seg-Lab v3+ algorithm developed in this article is a deep convolutional neural network with atrous convolution that achieves rapid and accurate identification of small target defects. The backbone of the network in Seg-Lab v3+ is YOLO v5s, which contributes to better feature characterization capabilities. The RASPP module is located on the right side of the Backbone Seg+ and employs multiple rate parallel atrous convolutions to extract features from different scales, achieve feature aggregation and reduce the number of channels with 1 × 1 convolutions. The FCAM module is used to combine Low-level Features with High-level Features in order to recover spatial information and provide clearer segmentation of feature information.

#### 2.3.1. Backbone Seg+ Module

**Figure 2:**
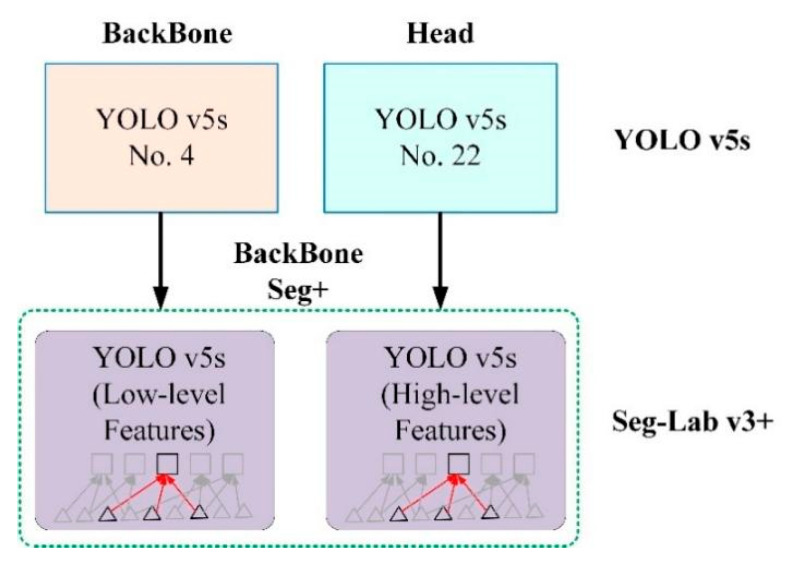
Backbone Seg+ module.

As shown in Figure 2, Backbone Seg+ is composed of Low-level features and High-level features. Low-level features have the characteristics of a large feature map and small perceptual field, which are suitable for representing some small detailed information in the image, such as the information of BigShavings and GlueSpots defects. The downsampling of High-level features increases and aggregates shallow features through bottom-up paths. The purpose is to fully fuse the image features of different layers with small feature maps and large perceptual fields, which are more suitable for representing the features of large-size defects in particleboard images, such as OilPollution, SandLeakage, and Soft.

#### 2.3.2. RASPP Module

**Figure 3:**
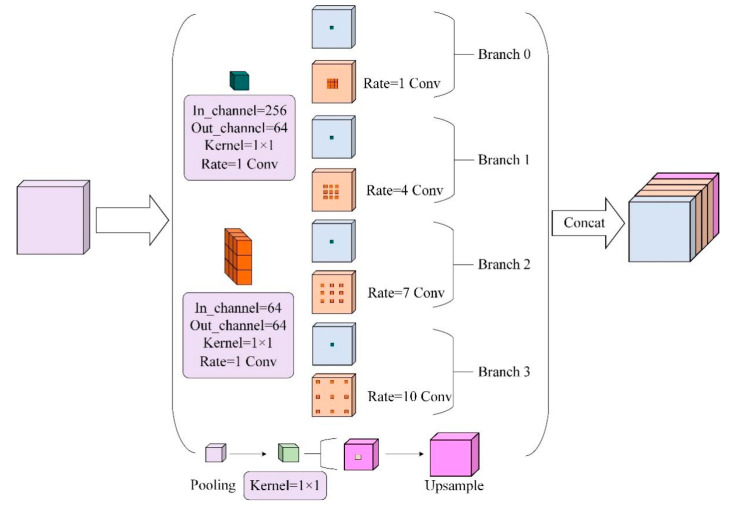
RASPP module.

As shown in Figure 3, the RASPP module is designed to extract multi-scale defect feature information by sampling atrous convolutions parallel with different expansion rates. This study adds independent 1 × 1 convolutions to the original ASPP module in order to reduce the computation time of the model, with the aim of reducing the number of input channels to the atrous convolution in the form of a bottleneck layer, and the module reduces the expansion rate of the atrous convolution from {6, 12 and 18} to {4, 7 and 10}. The RASPP utilizes the reduced atrous convolution for feature extraction and is fused together. The size of the receptive field is proportional to the size of the atrous convolutional expansion rate, reducing the receptive field and improving the accuracy of small target feature detection. Structure mining by the RASPP module reduces the loss of feature information for small target defects such as BigShavings and GlueSpot, and learns features more comprehensively.

#### 2.3.3. The FCAM Module (Feature Fusion Attention Mechanism)

**Figure 4:**
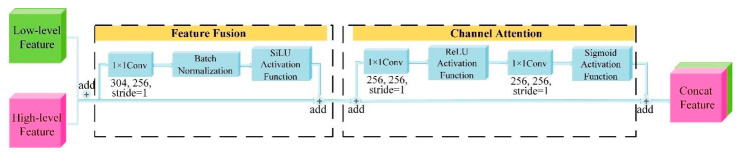
The FCAM module.

With the introduction of the Channel Attention structure in the Fusion part and the removal of the Dropout layer, this module aims to minimize the use of redundant features and minimize training time, as shown in Figure 4. To obtain more semantic information about features, FCAM combines Low-level Features and High-level Features. The channel attention is constituted by two 1*1 convolutional bottlenecks, with a ReLU activation function added after the first convolution and a Sigmoid activation function added after the second convolution. This project aims to speed up the training time of the network and solve the problem of gradient explosion. This method employs a channel-by-channel model to adjust the surface defect features’ channel-by-channel and global information to produce a more accurate selection of the defect features and suppress undesirable characteristics.

## 3. Materials and Methods

### 3.1. Data Collection and Pre-Processing

**Figure 5:**
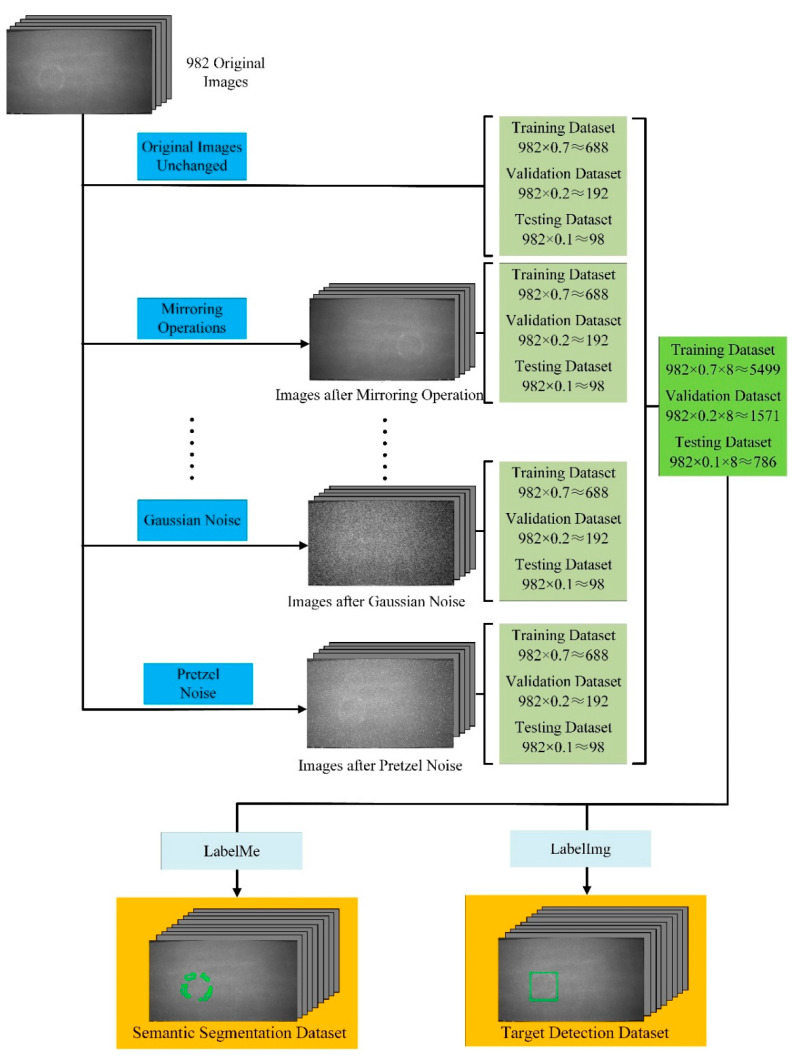
Data augmentation and annotatione.

The particleboard images were collected from the particleboard production facility of Fenglin Yachuang Group in Huizhou, China. The original picture size of the five different types of defective particleboard was 1751 by 911 pixels, with a bitmap depth of 8 bits, for a total of 982 images. These photos are split into three groups in a ratio of 7:2:1, and seven image modifications, including mirroring, grayscale conversion, median filtering, white noise, poisson noise, gaussian noise and pretzel noise are applied to each group. The dataset has 7856 total photos and is made up of both the enhanced data and the original photographs. Among them, 70% of the training set images are 982 × 0.7 × 8 ≈ 5499, 20% of the validation set images are 982 × 0.2 × 8 ≈ 1571 and the remaining 10% are used as the testing set images, which are 982 × 0.1 × 8 ≈ 786. This is shown in Figure 5.

Two programs, LabelImg and LabelMe, are used to generate target detection labels and semantic segmentation labels for five different types of defects seen on the surface of particleboard, including SandLeakage, BigShavings, GlueSpot, OilPollution, and Soft. Each area of interest is covered by the label, which also pinpoints the defective pixels. Table 1 provides details on the surface flaws in particleboard.

### 3.2. Training Hyper-Parameters

The following hardware configurations were used for training and testing: Intel^®^ Xeon^®^ Gold6152 CPU @ 2.10 GHz, 256 GB of RAM and NVIDIA Quadro RTX 8000 GPU. The software configuration is as follows: Windows Server 2012 R2 Standard, Python 3.8.3, CUDA version 10.1 and Pytorch dynamic development framework. The weights of the pre-trained model were initialized using YOLO v5s to exclude logit weights from the pre-trained model and fine-tuned in further training. The training hyperparameters used in this study were the optimal values chosen from multiple trials, as shown in Table 2.

### 3.3. Performance Evaluation Metrics

The performance of the model for particleboard surface defect recognition is evaluated by comparing it with real images. In this study, the model performance is evaluated by *precision*, *recall*, *F*_1_ *score*, *mAP*, and *mIoU*. The calculation equations are as follows:

(1) $$precision=\frac{TP}{TP+FP}$$

(2) $$recall=\frac{TP}{TP+FN}$$

(3) $$F_{1}score=\frac{2\times precision\times recall}{precsion+recall}$$

(4) $$mAP=\frac{\sum_{k=1}^{m}\int P_{k}(R_{k})dR_{k}}{m}$$

where *TP* represents the number of correctly identified defect targets; *FP* refers to the number of backgrounds erroneously identified as defect targets; *FN* indicates the number of undetermined defect targets; *P_k_* and *R_k_* represent *precision* and *recall* of the *k*th classification object, respectively; *M* represents the total number of objects detected in the dataset.

(5) $$mIoU=\frac{1}{m+1}\sum_{i=0}^{m}\frac{n_{ii}}{t_{i}+\sum_{j=0}^{m}n_{ji}-n_{ii}}$$

where *n_ij_* is the number of pixels for category *i* to be predicted as category *j*; *m +* 1 is the number of semantic segmentation categories (including background); $t_{i}=\sum_{j=0}^{m}n_{ji}$ is the total number of pixels for target category *i* (true labels); *n_ii_* is the number of pixels for category *i* to be predicted as a category *i* (the number of all correctly predicted pixels).

## 4. Results

Since the shallow feature layer output in the Backbone Seg+ module significantly impacts the accuracy of the detection of the model, this study conducted several trials and ultimately selected No.4 in the backbone network of YOLO v5s as the shallow feature layer output. The No.4 and No.19 in the YOLO v5s backbone network are YOLO v5-Seg-Lab-4 and YOLO v5-Seg-Lab-19.

### 4.1. Training Results

**Figure 6:**
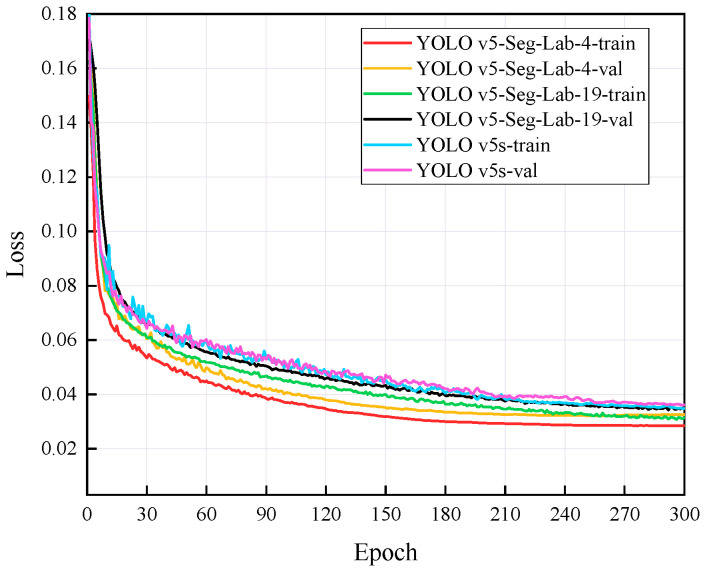
Comparison of loss functions under different scenarios.

The loss value represents the difference between the predicted value and the true value [40,41,42], and the size of the value inversely relates to the recognition effect of the predicted box. The training loss and validation loss for YOLO v5-Seg-Lab-4, YOLO v5-Seg-Lab-19 and YOLO v5s are depicted in Figure 6. With regard to convergence speed and the loss values experienced during training, the YOLO v5-Seg-Lab-4 model outperforms YOLO v5s. The final loss values for training and validation for YOLO v5-Seg-Lab-4 were 0.149 and 0.164, respectively, while the training and validation convergence losses for YOLO v5s were 0.178 and 0.182. The confidence score and predicted true class probability of each bounding box prediction increased significantly as the loss value of the YOLO v5-Seg-Lab-4 model decreases. It was found that YOLO v5-Seg-Lab-4 displayed a high rate of convergence during training for 30 epochs, which decreased between 30 and 90 epochs, and the loss curve converged after 150 epochs.

**Figure 7:**
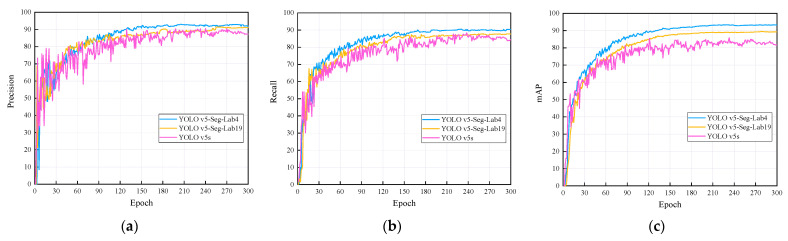
Comparison of evaluation indexes under different models. (**a**) precision curves, (**b**) recall curves, (**c**) mAP curves.

As illustrated in Figure 7, the precision, recall and mAP curves of the algorithm in this paper have been compared with those of the YOLOv5s network, with a higher value for each metric indicating a higher performance of the model. During the training process, the evaluation index curve of the algorithm presented in this paper exhibits a flat rise. Four evaluation indexes of the YOLO v5-Seg-Lab-4 are higher than those of the YOLO v5-Seg-Lab-19 and YOLO v5s. A total of 92.15, 91.08% and 89.04% were for precision, 93.38, 87.76% and 86.93% for recall, and 93.32, 89.27% and 85.53% for mAP, respectively. Since shallow feature layers are responsible for identifying areas with large targets, they require a small receptive field. Therefore, the YOLO v5-Seg-Lab-19 model has a large receptive field, which results in decreased model detection.

### 4.2. Recognition Results of YOLO v5-Seg-Lab-4

**Figure 8:**
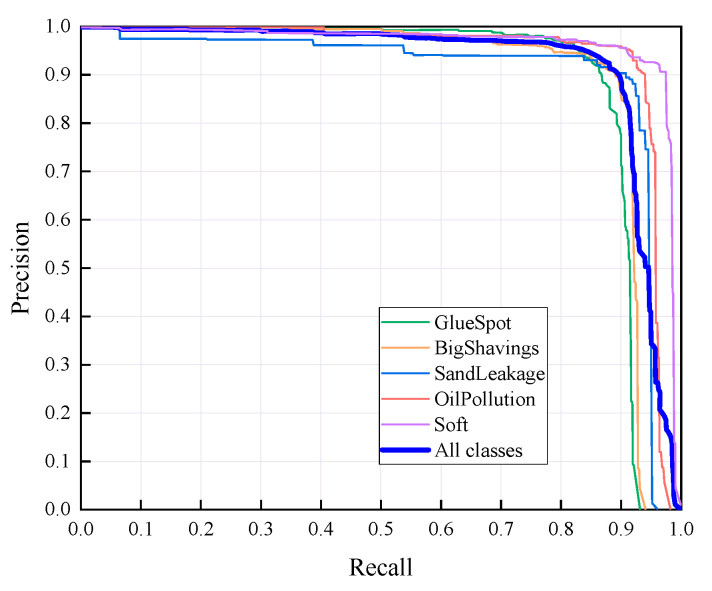
P-R curves.

**Figure 9:**
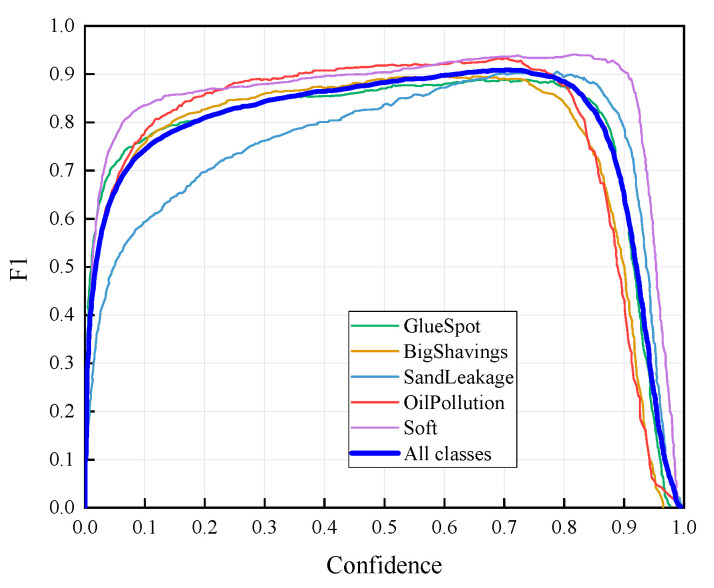
F_1_ Score curves.

According to Figure 8 and Figure 9, the recall accuracy curves and F_1_ score curves are plotted for the five particleboard surface defects included in the test dataset, with the area occupied by the curves proportional to the model detection effect. As a result, Soft and OilPollution occupy the largest areas, with easy identification due to their homogeneous geometry, the significant differences in gray scale values between these defects and other defects, and the low level of misjudgment rate that are observed. Furthermore, due to the random nature of the sizes of BigShavings, GlueSpot and SandLeakage in the production line, the recognition rate found in the dataset is lower than that of Soft and OilPollution. Grayscale values between SandLeakage panels and particleboard panels are not significantly different, which may make the recognition more difficult.

The results of the YOLO v5-Seg-Lab-4 model for the detection of five particleboard flaws were assessed using four evaluation metrics, including accuracy, recall, F1 score, and mAP, with a correct average rate of 92.4%, 89.8%, 91.1% and 93.2%, respectively, as shown in Table 3. In this instance, mAP is a crucial indicator of the effectiveness of defect detection. It is the sum of the average accuracy of all categories divided by all categories. The findings demonstrate that the YOLO v5-Seg-Lab-4 model performs superbly in detecting particleboard defects, particularly for Soft, with mAP results reaching 97.9%. With mAP results reaching 94.9%, the OilPollution detection impact is likewise excellent. The correct detection rates of the other three defects are above 90%, indicating that the YOLO v5-Seg-Lab-4 model, with the introduction of the attention mechanism, has an excellent performance in extracting particleboard defect information and improving defect detection accuracy under complex scene conditions.

### 4.3. Comparison of Different Algorithms

In this paper, the currently popular deep learning algorithms were applied to particleboard surface defects; the representative algorithms included YOLO v5s, YOLO v3, YOLO v4, DeepLab v3+ (MobileNet), DeepLab v3+ (Xception) and U-Net. By default, the weight file with the best training effect is saved as the weight file, which is used at testing time. The experimental results are shown in Table 4 and Table 5. The main information involved in the comparison includes model mAP value, mIoU value, single-image recognition time, number of parameters and FPS.

Compared with the YOLO v5s, YOLO v3 and YOLO v4 model, the mAP of the proposed YOLO v5-Seg-Lab-4 model has improved by 2.4%, 5.6% and 12.5%, respectively, and it is a significant improvement. The sensitivity of the SELayer-enhanced model to channel and spatial features was added. The features of useful information and suppression of useless features were emphasized to improve the adaptive ability of the model to the receptive field. Moreover, which mainly benefits SELayer by light architecture, the remarkable improvement in the inspection performance of the YOLO v5-Seg-Lab-4 does not sacrifice the detection speed. The average recognition speed of YOLO v5-Seg-Lab-4 is 56.02 fps (frames per second), and the average detection speed of each image is 17.85 ms, which is 3.95 and 5.02 times higher than the network efficiency of YOLO v4 and YOLO v3, respectively, which shows the potential of YOLO v5-Seg-Lab-4 in the case of limited hardware.

The mIoU of the YOLO v5-Seg-Lab-4-based approach was 5.72%, 3.98% and 10.66% higher in segmentation as compared to DeepLab v3+ (MobileNet), DeepLab v3+ (Xception) and U-Net. The average inference time for each image for the other three models was almost 14.39, 11.95 and 10.19 times slower than YOLO v5-Seg-Lab-4. This suggests that YOLO v5-Seg-Lab-4 is more favorable when high precision and real-time are required. Moreover, which mainly benefits RASPP and FCAM by innovating, the remarkable improvement in the small target defect inspection performance of the YOLO v5-Seg-Lab-4 does mean network detection time is accelerated.

On the original image, the mask map obtained from testing was applied to achieve the segmentation results. Since the particleboard surface defects are in the form of small targets, the defective ROI portion of the segmentation results is intercepted, as shown in Table 6. The table shows that the segmentation map generated by YOLO v5-Seg-Lab-4 closely matches the ground truth image, and the defect features extracted from the image are considerably enhanced. The fused attention mechanism module in the network is able to focus on pixel points with small target defects, thus filtering out complex interference information from the particleboard surface. Meanwhile, it increases the efficiency and accuracy of the calculation of the model, and it indicates with greater accuracy the distribution position of defects on the particleboard surface with good segmentation results. However, DeepLab v3+ (MobileNet), DeepLab v3+ (Xception) and U-Net failed to capture the edges of small target defects during segmentation. Due to the high similarity between the background of the particleboard and the grayscale values of SandLeakage, a higher percentage of defective pixels were lost from the U-Net. Furthermore, some of the smaller regions in the table that represents defect are not adequately captured by the segmentation models other than YOLO v5-Seg-Lab-4.

## 5. Discussion

**Figure 10:**
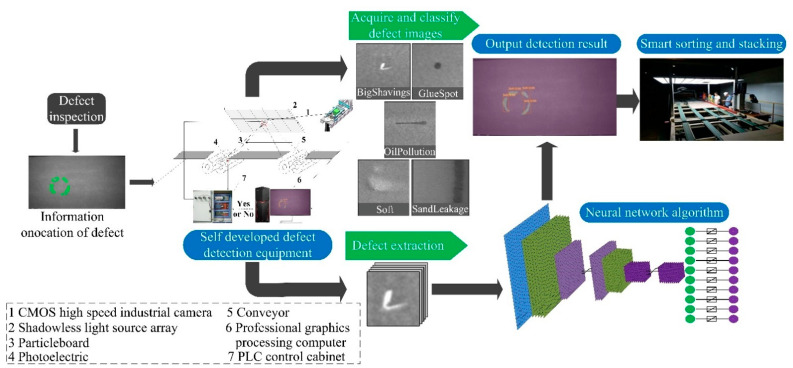
Particleboard surface defect detection system.

The YOLO v5-Seg-Lab-4 algorithm as a surface defect detection method is applied to particleboard continuous press generation equipment, which is located in Huizhou Fenglin Yachuang particleboard plant, as shown in Figure 10. Particleboard is moved along the production facility’s conveyor belt at a speed of 1500 mm/s. The JAI SP-5000M-CXP4-USB 3.0 camera is mounted above the conveyor belt, along with a series of LED lights that emit uniform light. The photoelectric sensor is activated when the chipboard moves beneath the light, and the camera then records an image of the chipboard’s surface. The algorithm marks defects in the image and uses them for particleboard defect analysis and categorization determination.

The system was run continuously for 8 h, during which time 8762 particleboards were inspected, each for between 183 and 208 milliseconds, thus meeting the real-time needs of the production line. However, manual inspection takes more than 2000 ms per particleboard, which is nearly ten times slower than the real-time detection of this research method. The detection rates of surface defects for the five particleboard types are shown in Table 7. Because the particleboard surface was sanded unevenly, the gray values in the under-sanded areas in the middle were significantly lower than the gray values in the surrounding over-sanded areas, leading to the system mistaking low gray values for defects.

**Figure 11:**
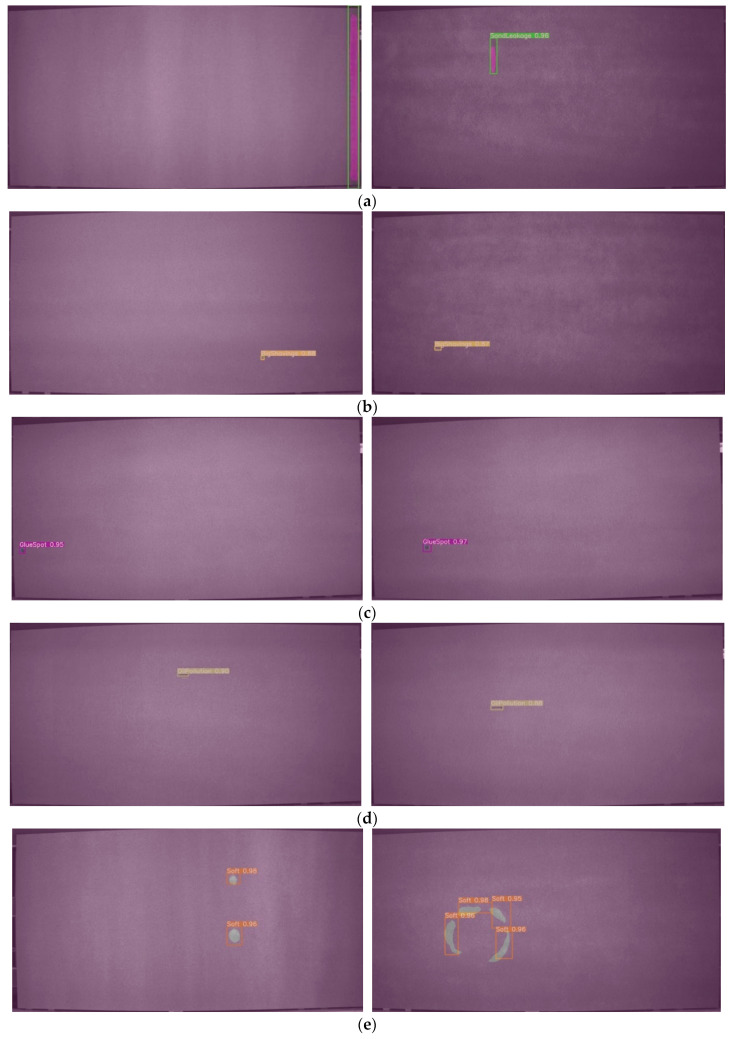
Image of the results of particleboard surface defect detection. (**a**) SandLeakage defects, (**b**) BigShavings defects, (**c**) GlueSpot defects, (**d**) OilPollution defects, (**e**) Soft defects.

Results of the field tests are presented in Figure 11, where both Soft and GlueSpot detected results between 0.95 and 0.98. Meanwhile, OilPollution, BigShavings, and SandLeakage, although located near the edges of the particleboard, were all accurately identified, unaffected by the size, grayscale unevenness, etc., with detection results between 0.88 and 0.95. The results suggest that the proposed model in this paper can locate small target defects accurately, and the task of determining the defect type and binning can be completed within the specified time for each particleboard. Moreover, the model achieves a lightweight application on mobile devices, meeting the practical needs of particleboard defect detection.

## 6. Conclusions

According to the characteristics and application requirements of particleboard surface defects, this paper puts forward the defect detection method of YOLO v5-Seg-Lab-4. YOLO v5-Seg-Lab-4 uses FCAM-based fusion of deep and shallow features to recover spatial information and produce sharper segmentation features, and the feature aggregation results in the advantage of multi-rate parallel atous convolution to extract features from different scales. This aforementioned method improves the adaptability of the receptive field, enhances the accuracy and efficiency of five kinds of defect detection, and realizes the detection, segmentation and marking of small target defects on the surface of large-format particleboards. The YOLO v5-Seg-Lab-4 algorithm, as a surface defect detection method, has been put into use in the Huizhou particleboard factory, realizing the classification and board grading of SandLeakage, BigShavings, GlueSpot, OilPollution and Soft. The defect detection accuracy rate reaches 98.2%, the time required for detection is between 183 ms and 208 ms, and the particleboard detection is accurate and efficient. In the future, the method is required to be expanded to several particleboard manufacturers and the algorithm optimized according to the usage environment to solve the problem of particleboard surface defect detection and segmentation in more complex scenarios.

## Figures and Tables

**Table 1:** Specific information of particleboard surface defect dataset.

Serial Number	Classification Type	Defect Characteristics	The Grayscale Amplitude of the Image	Interpretation Features	Total Number of Defects after Augmentation
1	SandLeakage	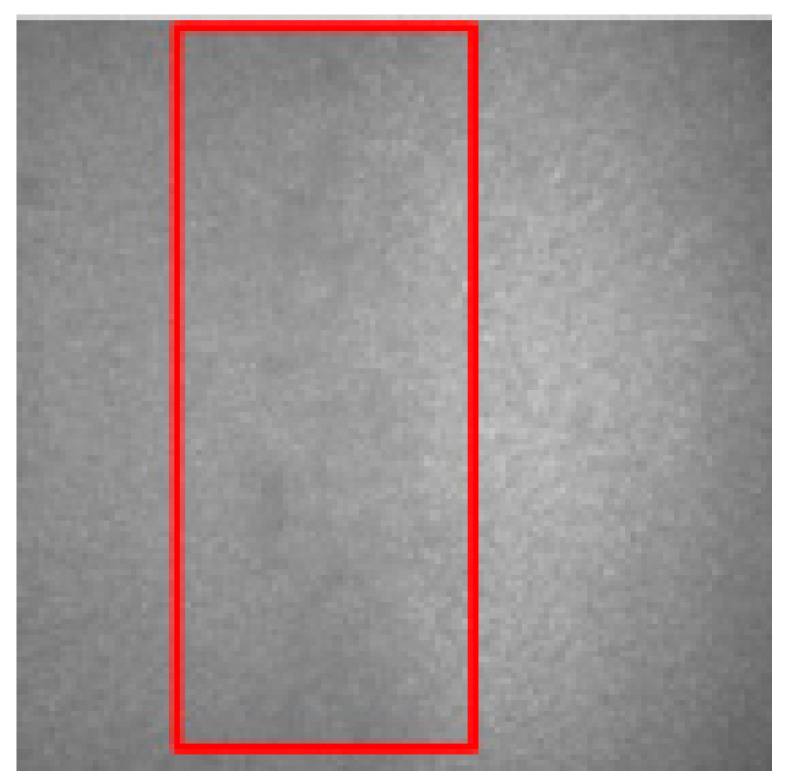	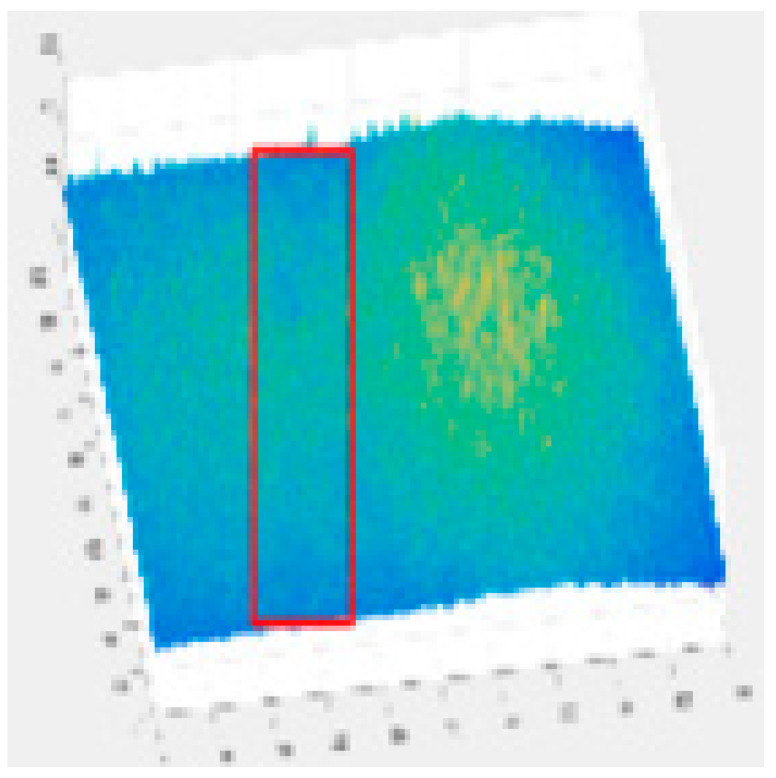	Dark gray, large area, a gray value close to the board gray value, difficult to identify.	809
2	BigShavings	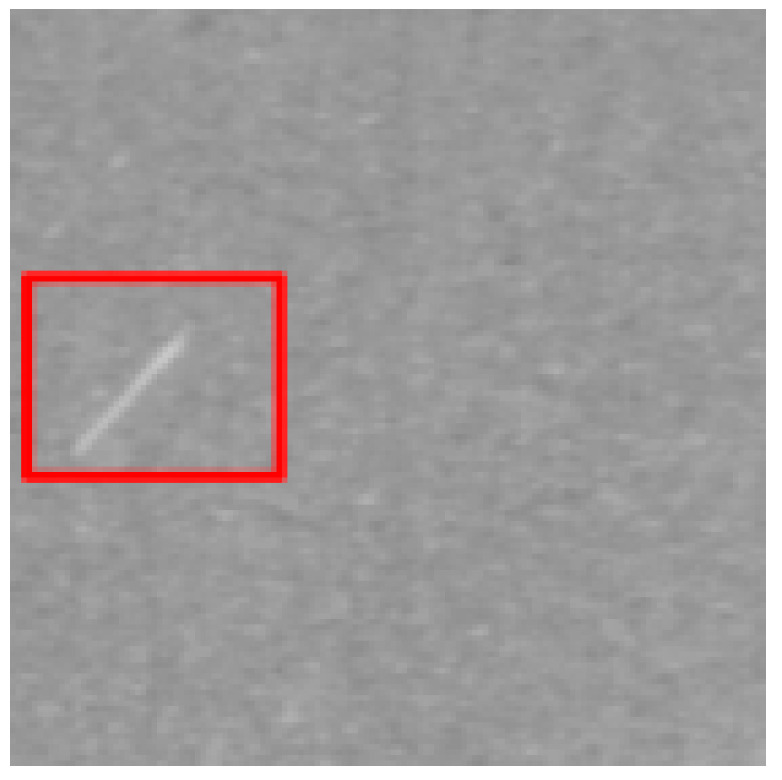	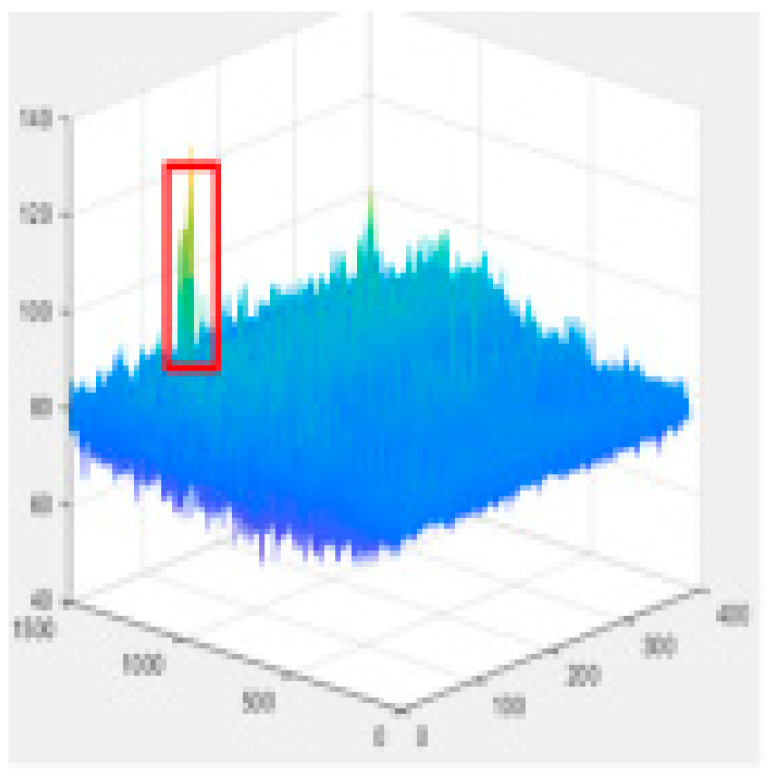	White thin strip, small size, the large gap between gray value and board gray value.	2021
3	GlueSpot	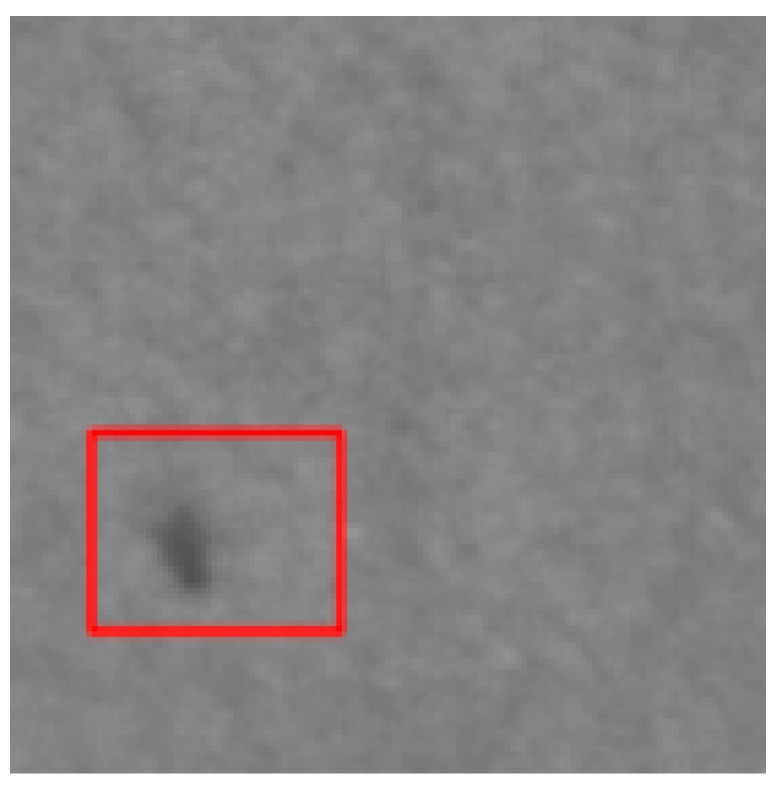	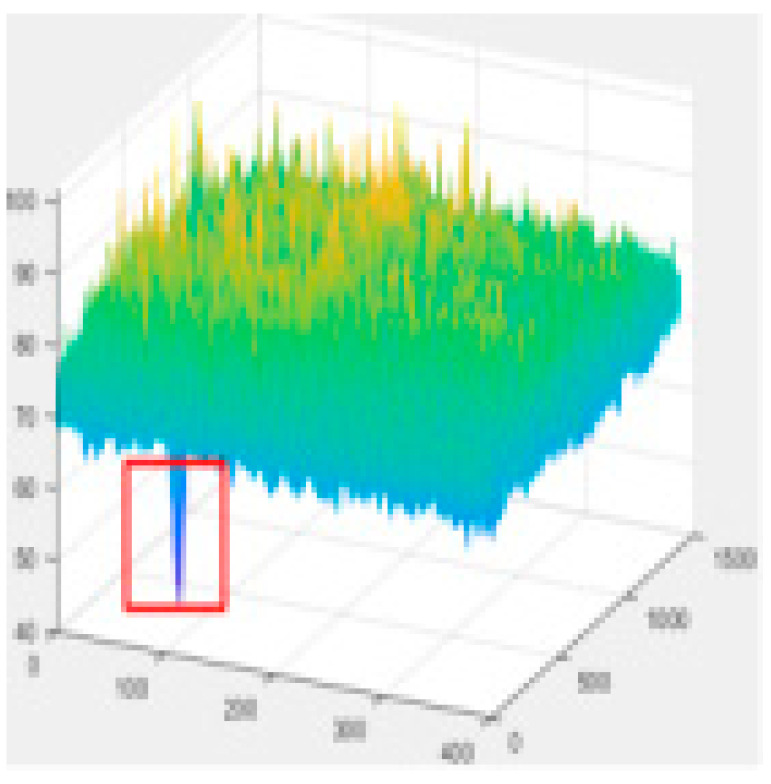	Black, round and spherical, small in size, the large gap between gray value and board gray value.	2736
4	OilPollution	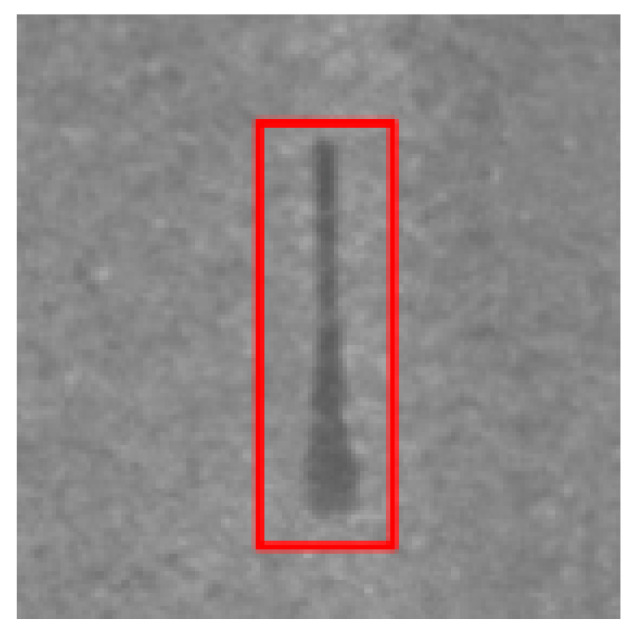	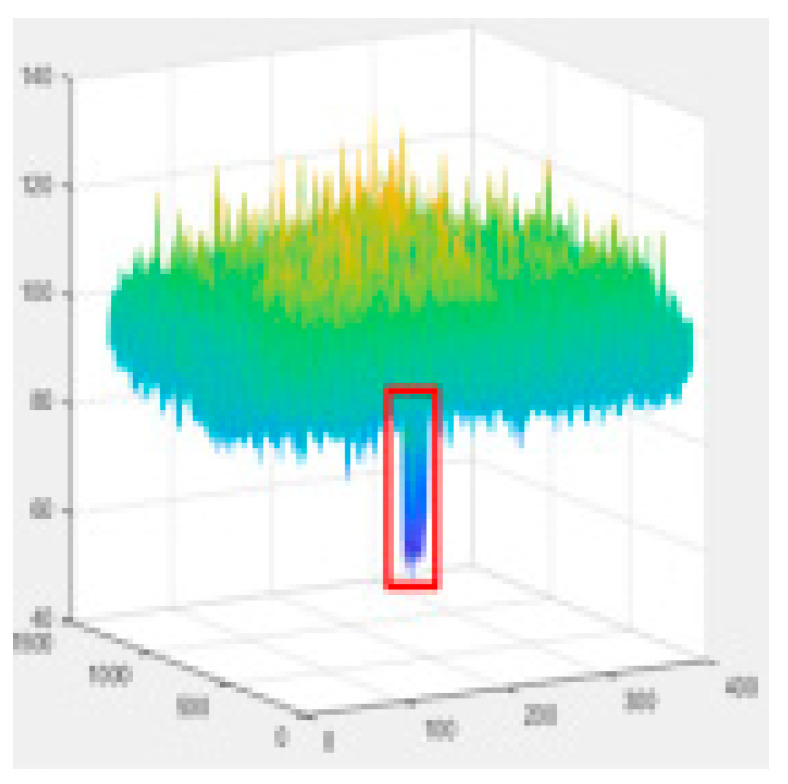	Black, wide strip, the large gap between gray value and board gray value.	1310
5	Soft	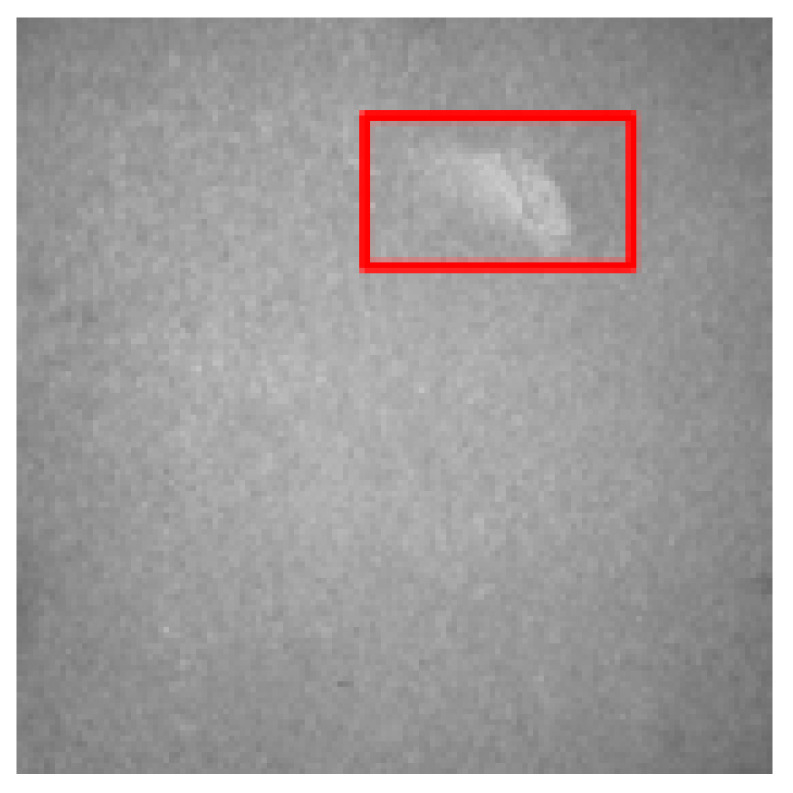	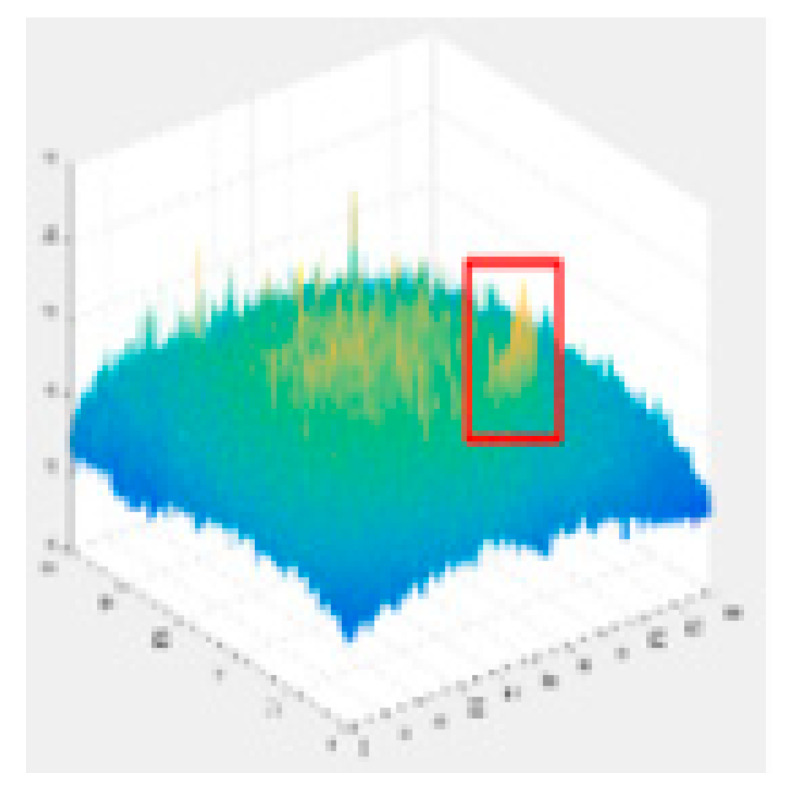	Dark gray and white, gray value and board gray value similar, difficult to identify.	3526

**Table 2:** Training hyper-parameters used in this model.

Training Parameters	Values
Initial learning rate	0.0015
Final learning rate	0.2
Optimizer	Adam
Momentum	0.937
Weight decay	0.0005
Train batch size	32
Epochs	300

**Table 3:** Identification results of the model for particleboard surface defect targets in this paper.

Class	Precision (%)	Recall (%)	F_1_ Score (%)	mAP (%)
SandLeakage	88.7	92.5	90.6	92.1
BigShavings	93.6	84.3	88.7	90.5
GlueSpot	93.5	84.5	88.8	90.8
OilPollution	95.5	90.1	92.7	94.9
Soft	90.5	97.8	94.0	97.9
All	92.4	89.8	91.1	93.2

**Table 4:** Performance comparison of five object detection networks.

Model	mAP (%)	Single-Image Recognition Time (ms/pic)	FPS	Number of Parameters	Size of Model (MB)
YOLO v5-Seg-Lab-4	93.2	17.85	56.02	7,952,864	15.9
YOLO v5-Seg-Lab-19	89.3	18.91	52.88	7,959,008	15.9
YOLO v5s	90.8	17.63	56.72	7,257,790	14
YOLO v4	87.6	70.36	14.21	63,913,079	244
YOLO v3	80.7	89.63	11.16	61,528,501	235

**Table 5:** Performance comparison of five semantic segmentation networks.

Model	mIoU	Single-Image Recognition Time (ms/pic)	FPS	Number of Parameters
YOLO v5-Seg-Lab-4	0.7663	17.85	56.02	7,952,864
YOLO v5-Seg-Lab-19	0.7244	18.91	52.88	7,959,008
DeepLab v3+ (MobileNet v2)	0.7248	181.81	5.51	2,753,714
DeepLab v3+ (Xception)	0.7369	213.22	4.69	4,253,330
U-Net	0.6925	265.96	3.76	31,032,837

**Table 6:** Comparison of defect segmentation result on test dataset.

Class	Origin Image	Ground Truth	YOLO v5-Seg-Lab-4	YOLO v5-Seg-Lab-19	DeepLab v3+ (Xception)	DeepLab v3+ (MobileNet v2)	U-Net
Sand-Leakage	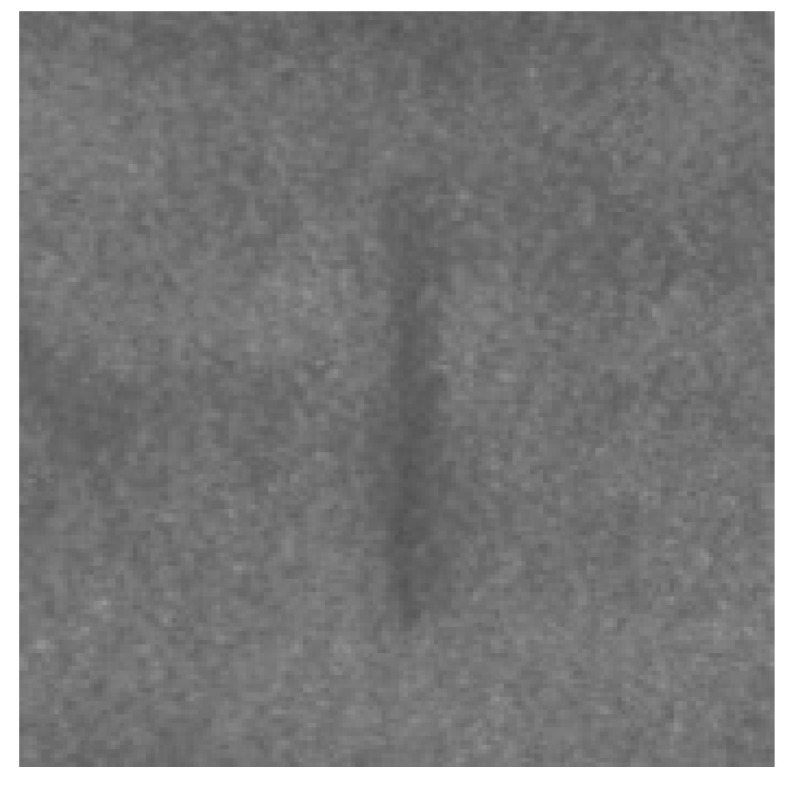	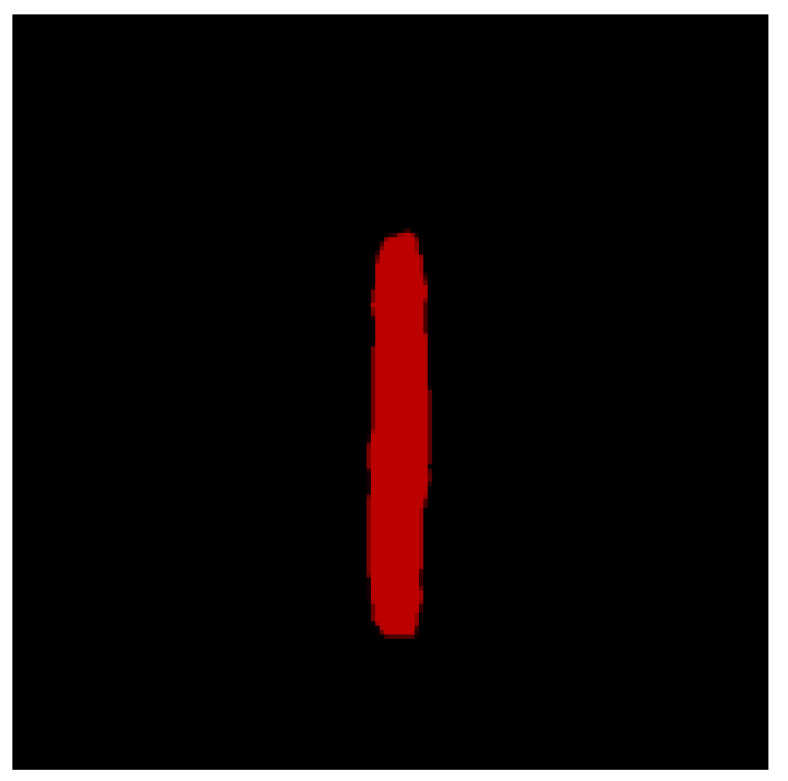	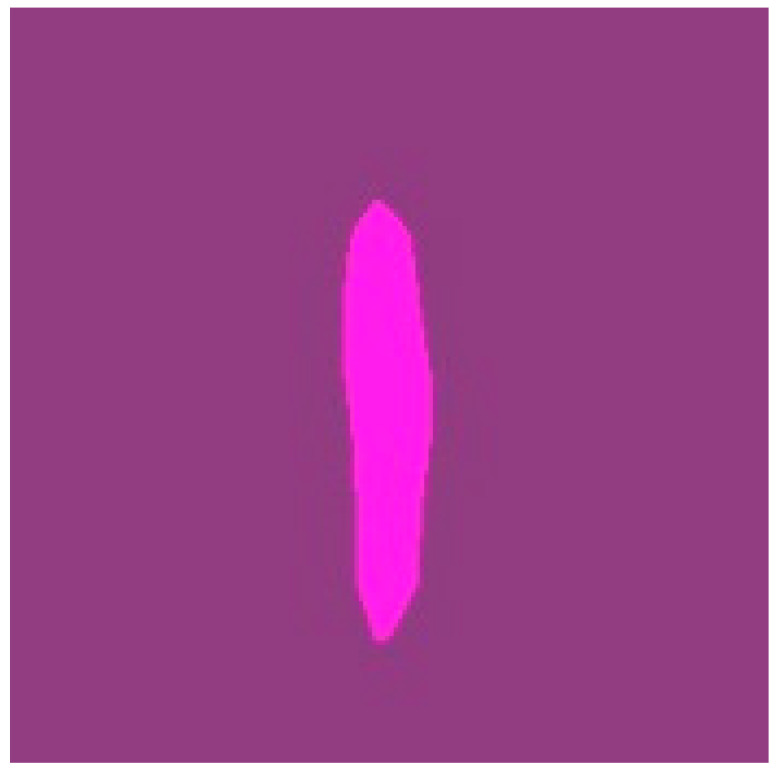	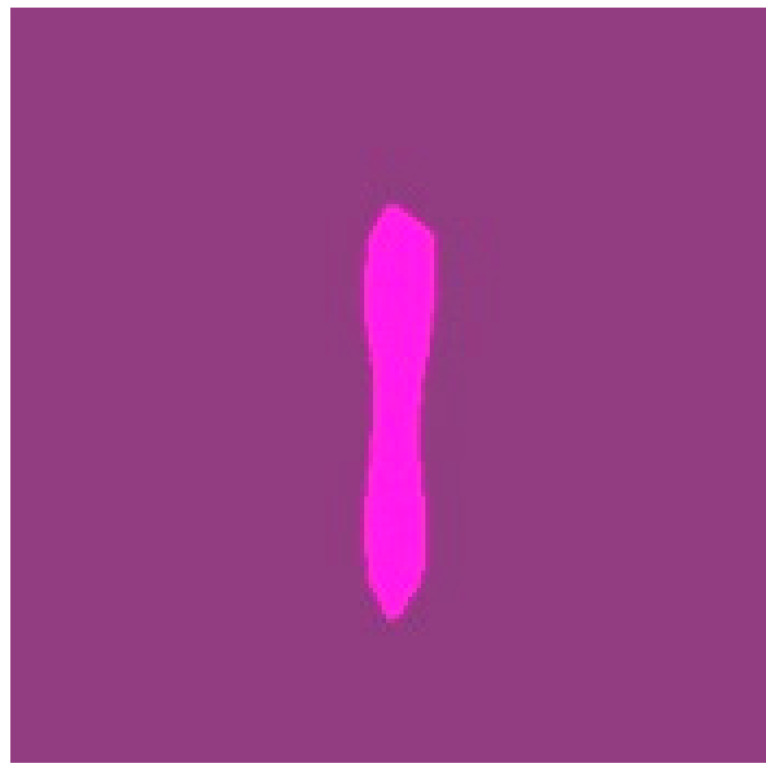	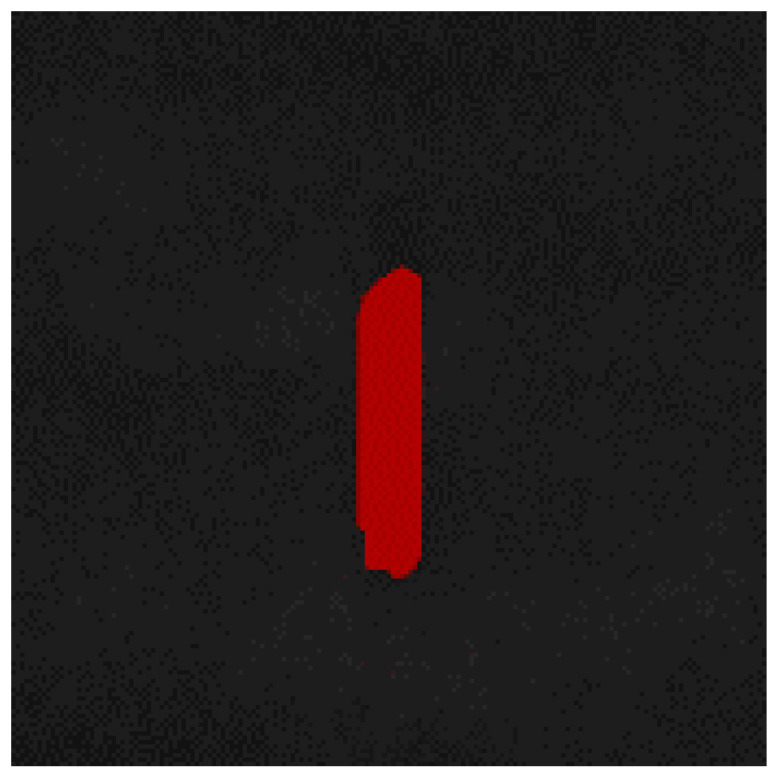	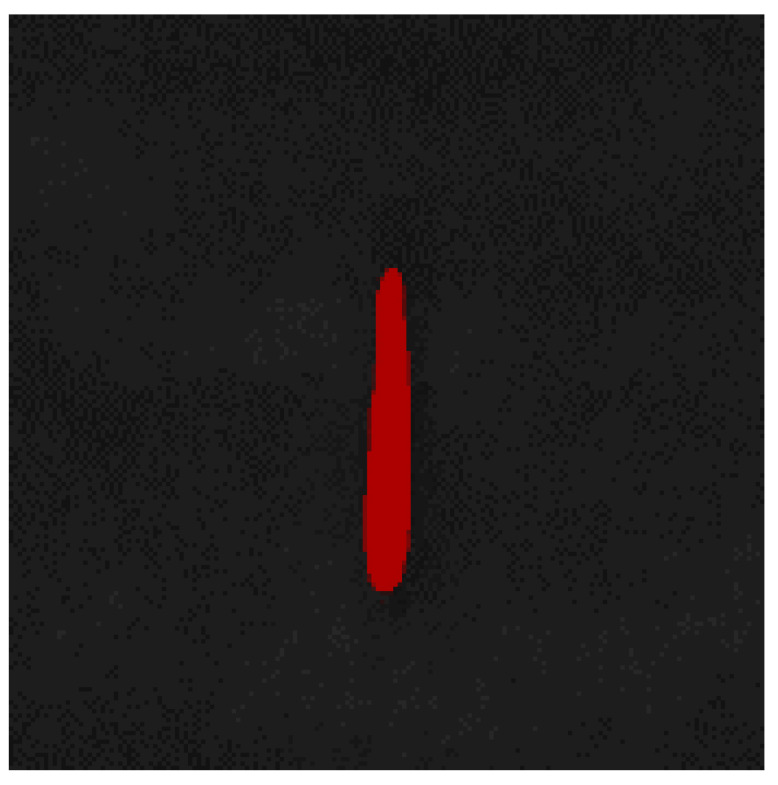	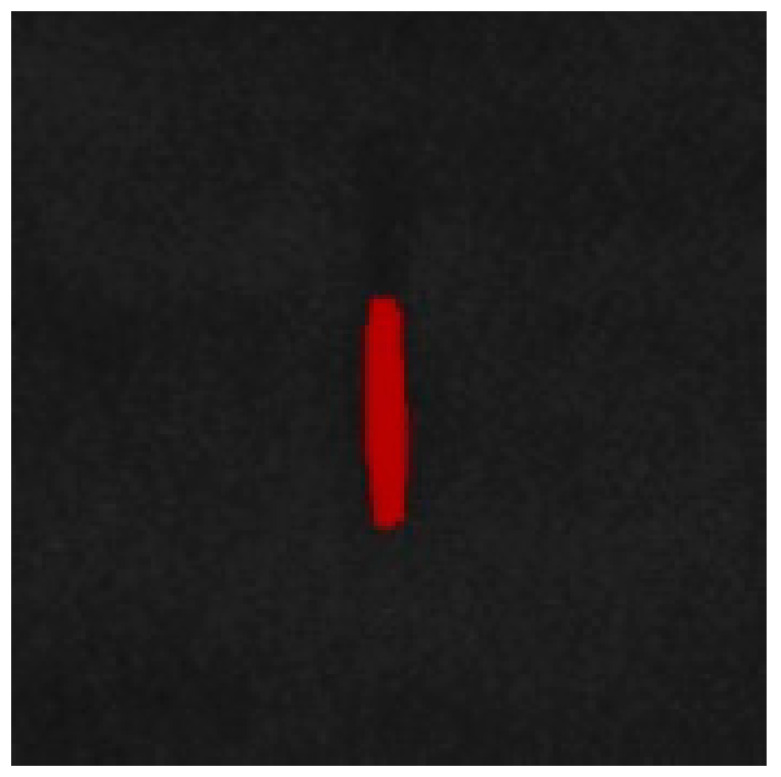
Big-Shavings	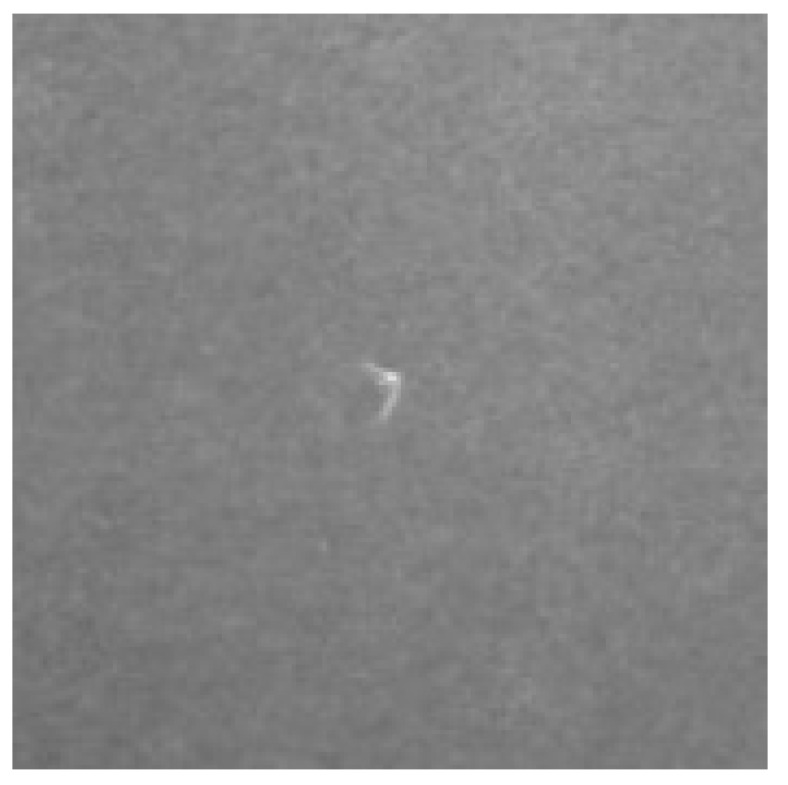	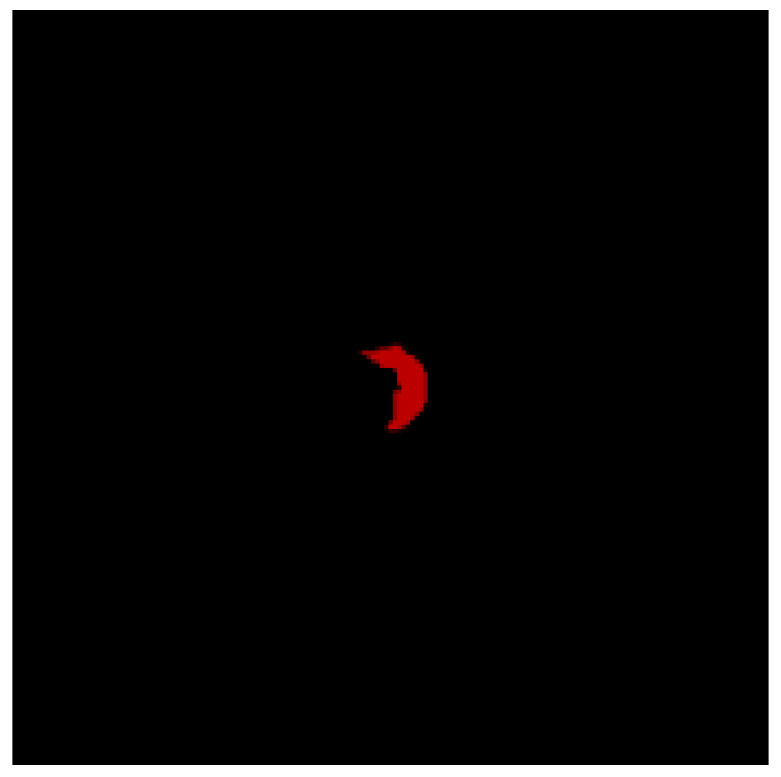	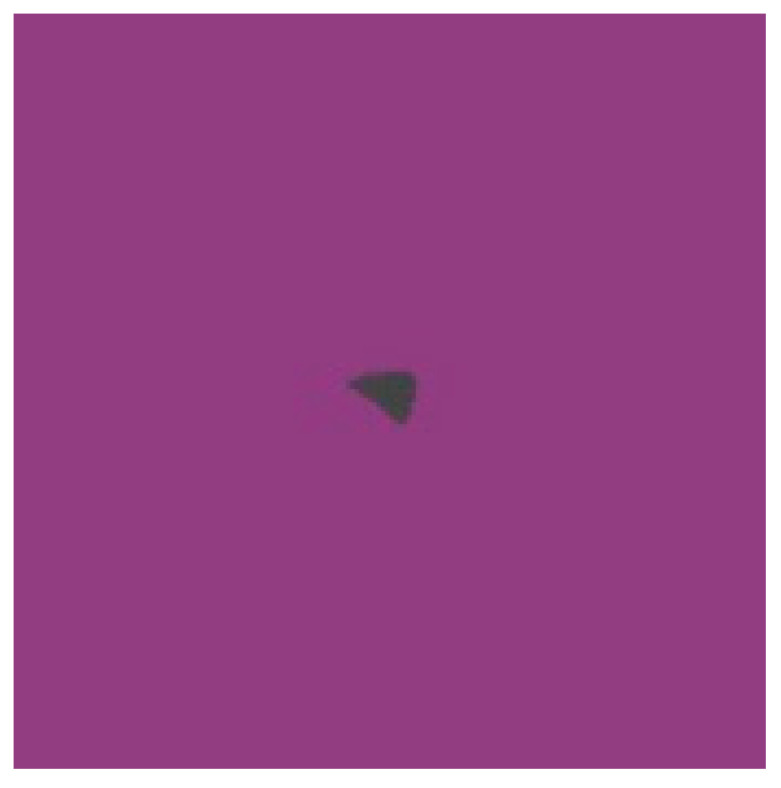	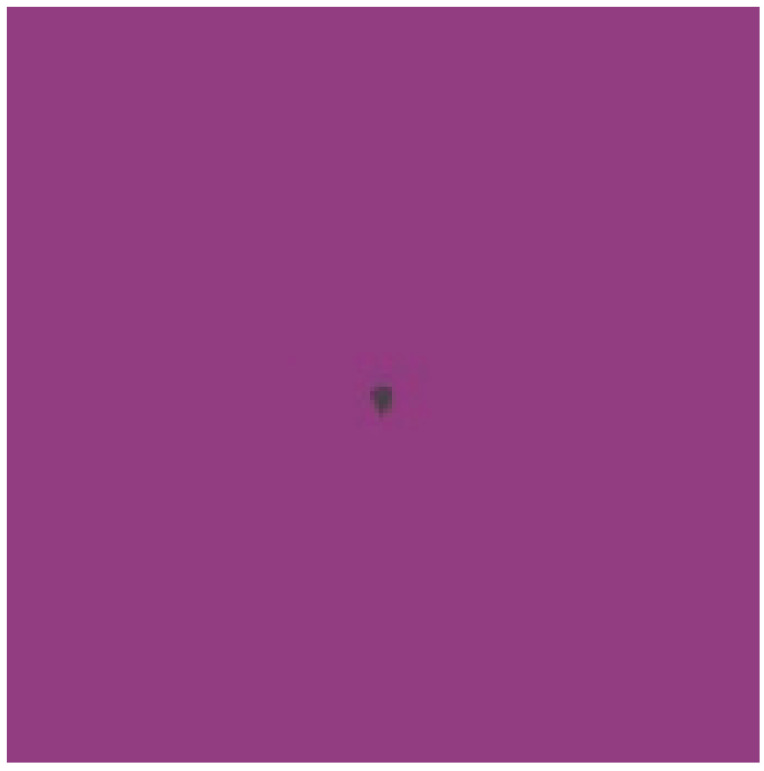	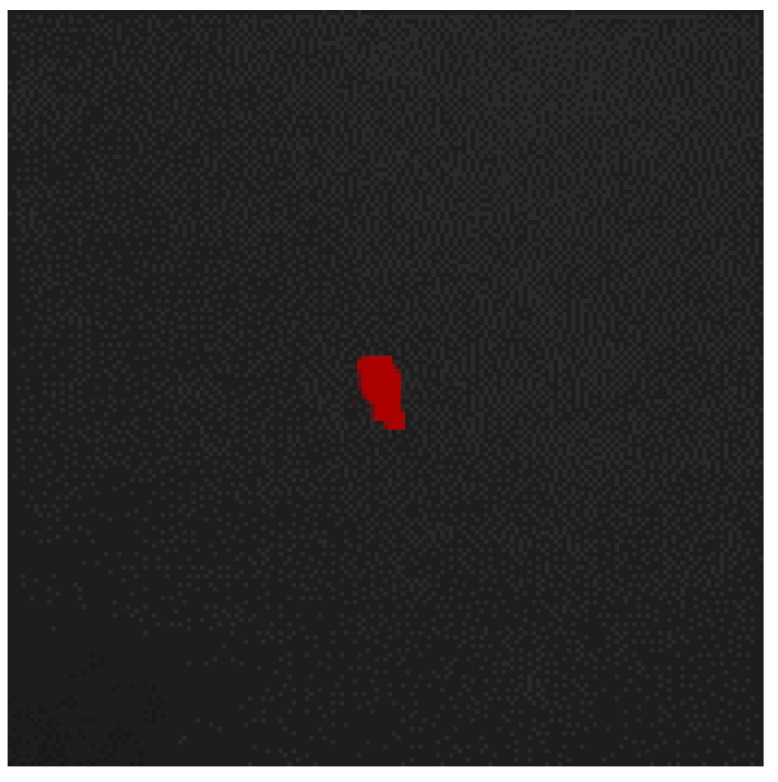	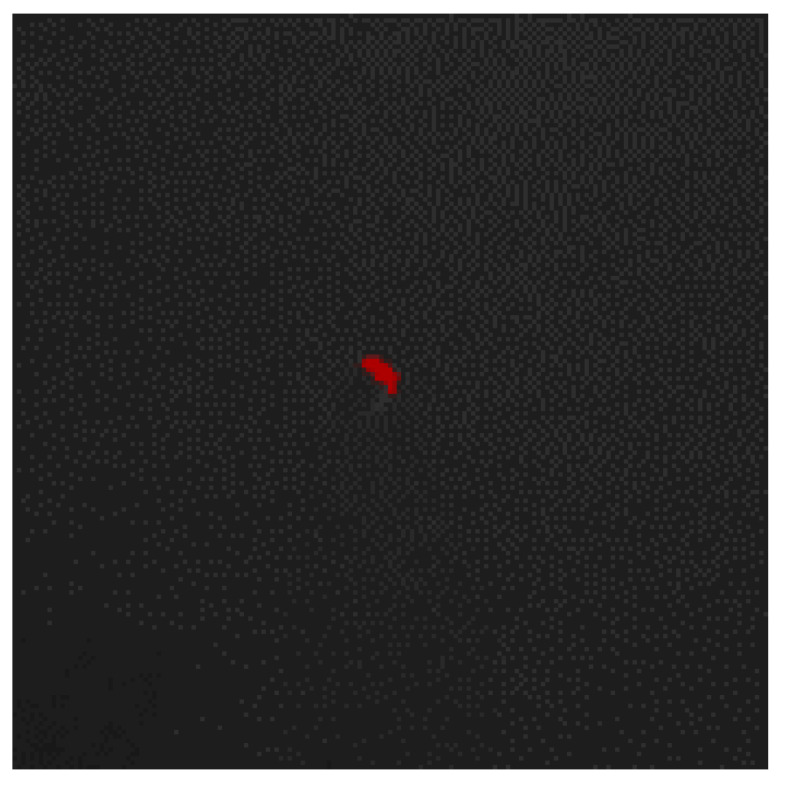	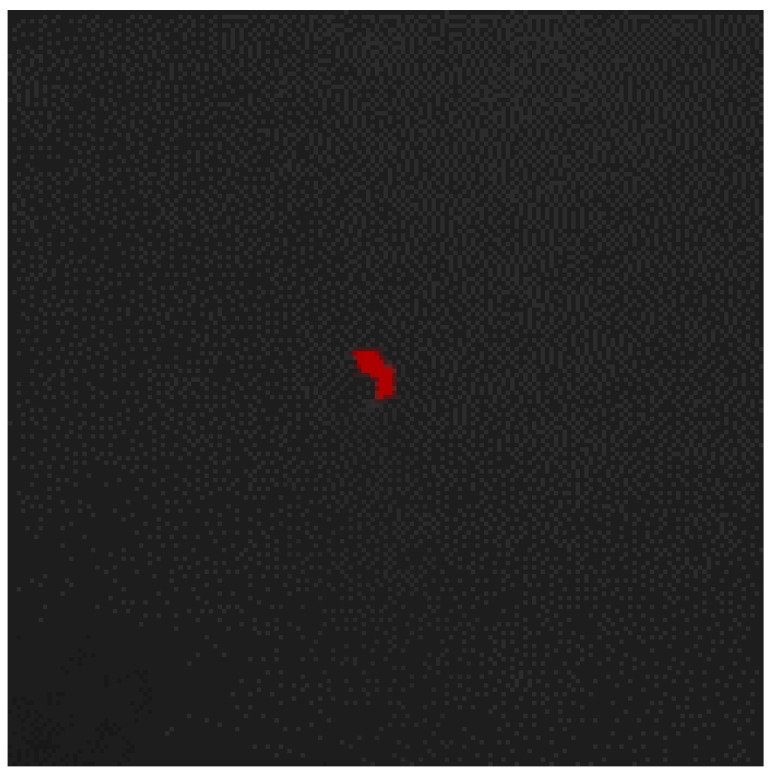
GlueSpot	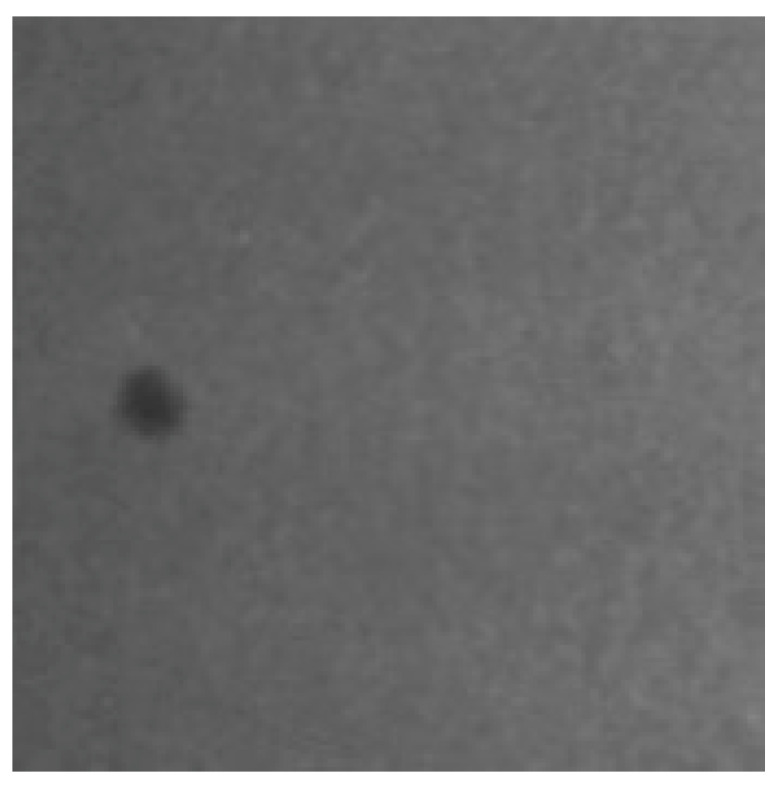	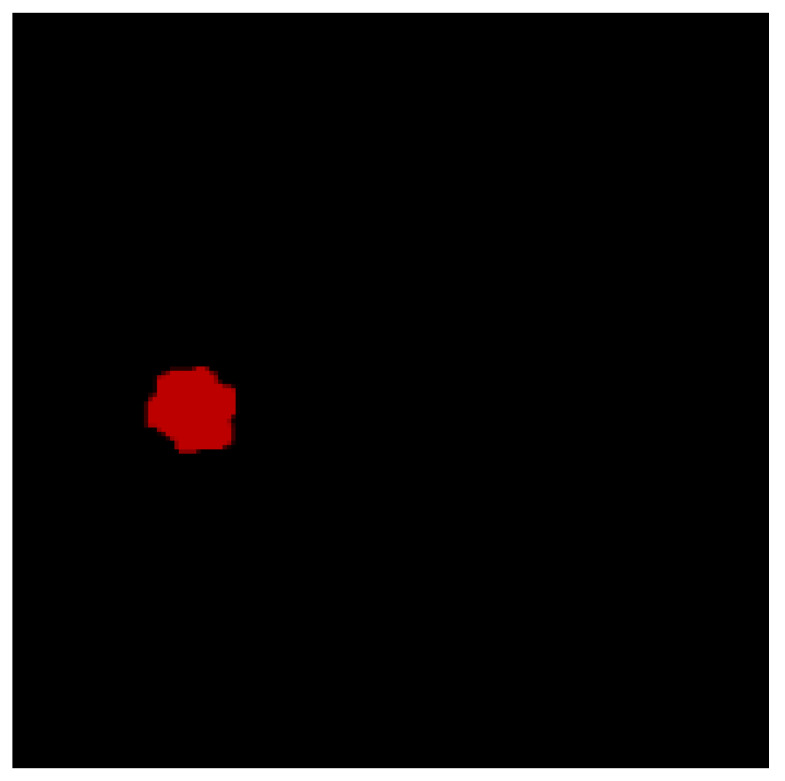	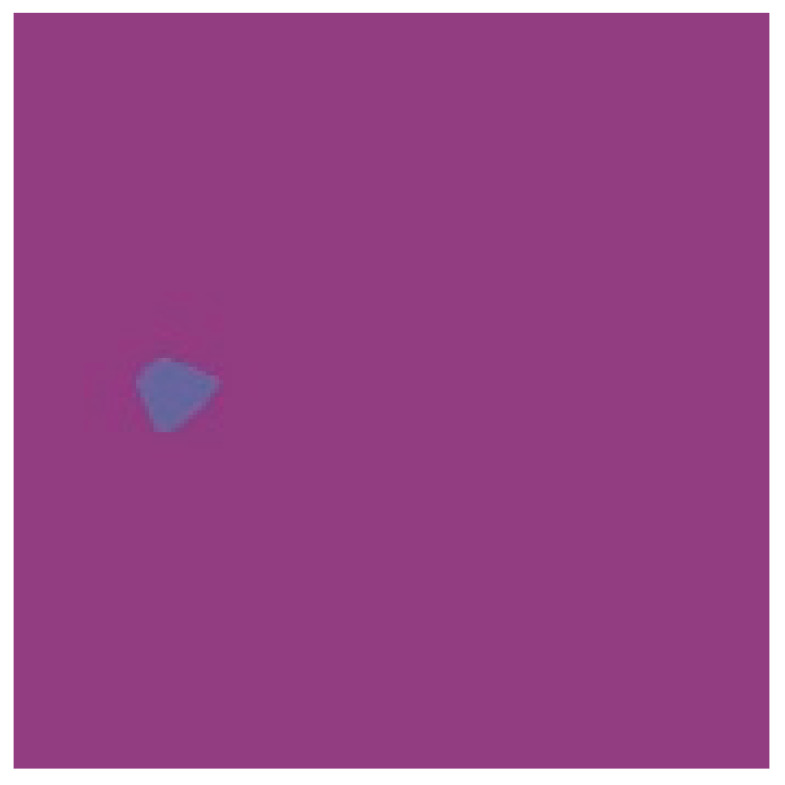	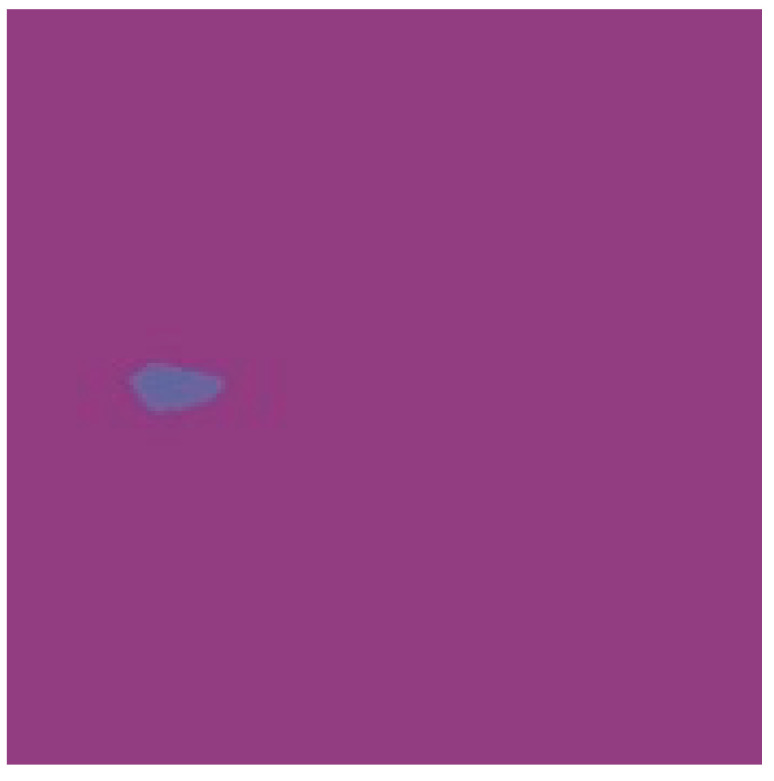	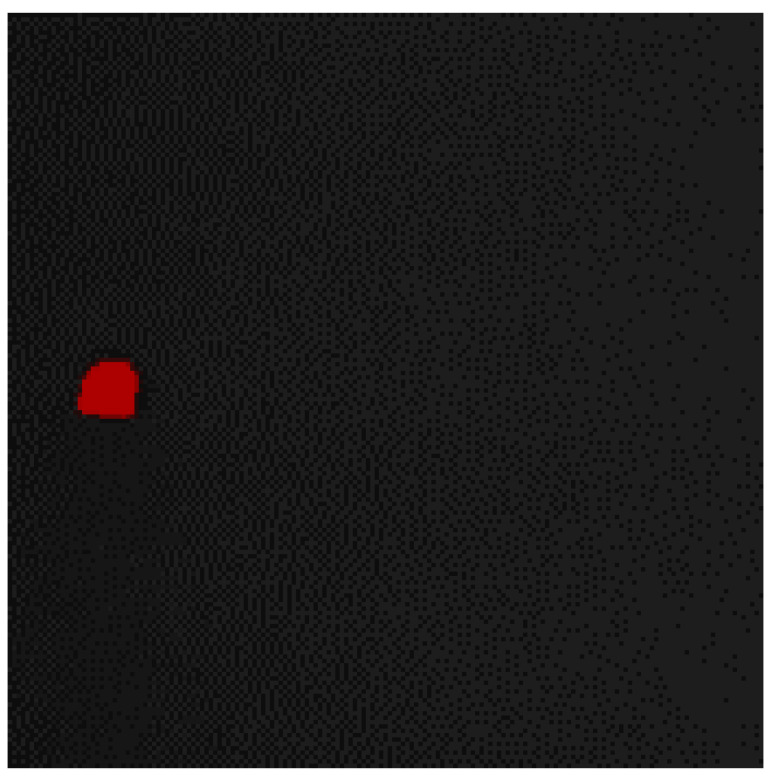	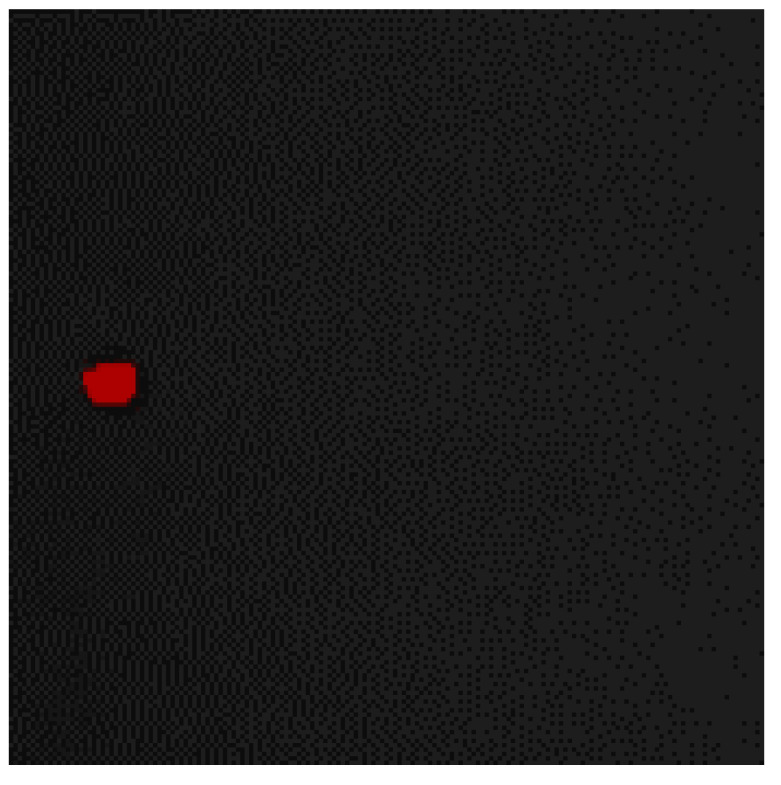	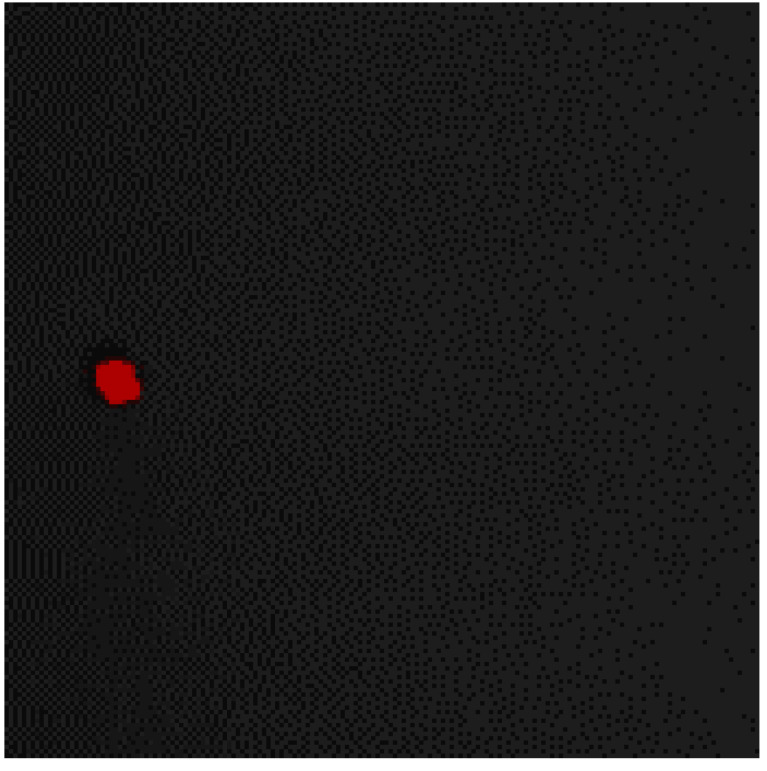
Oil-Pollution	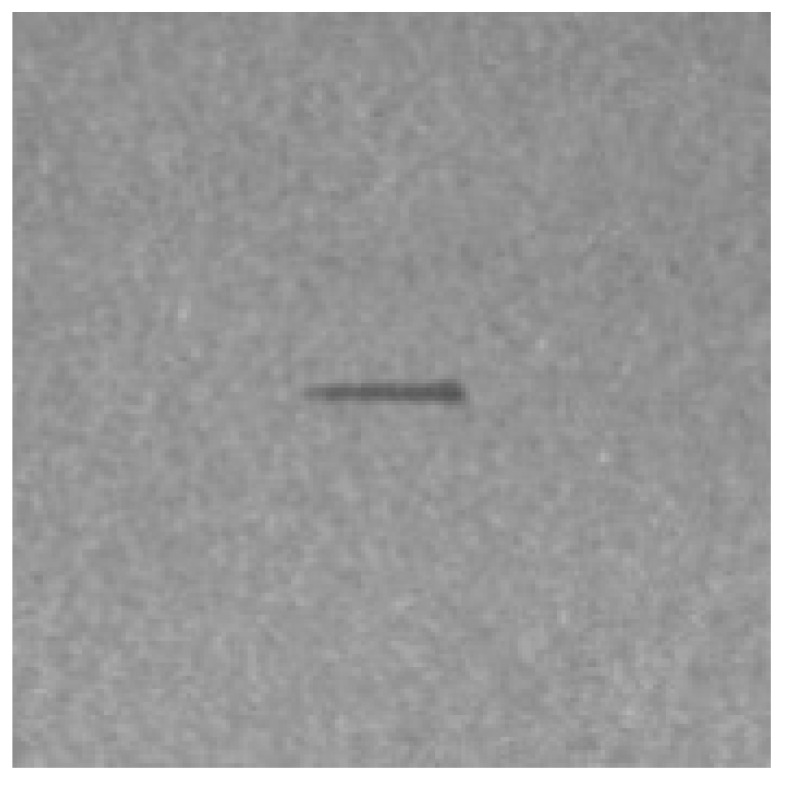	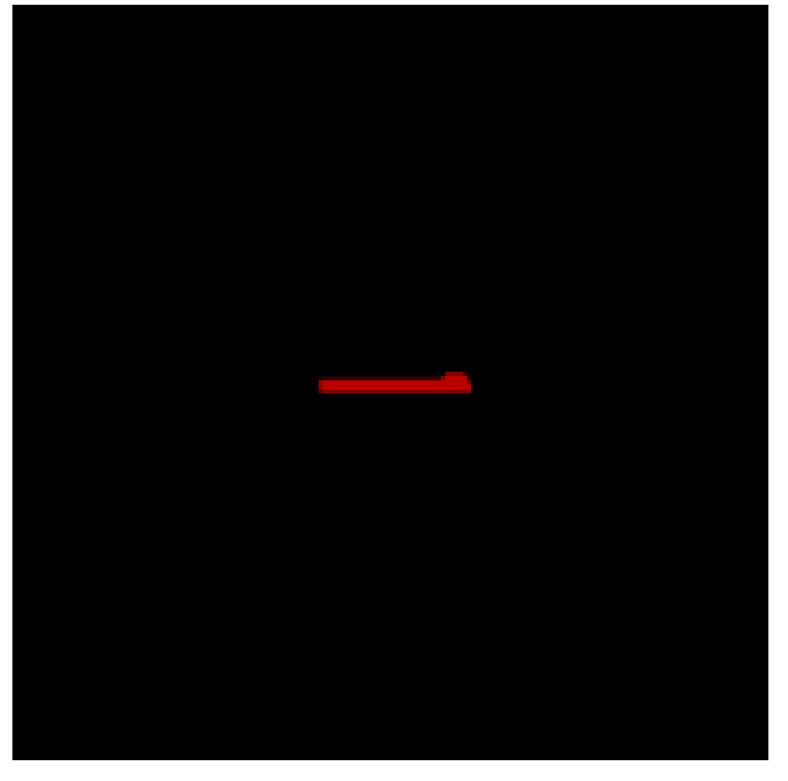	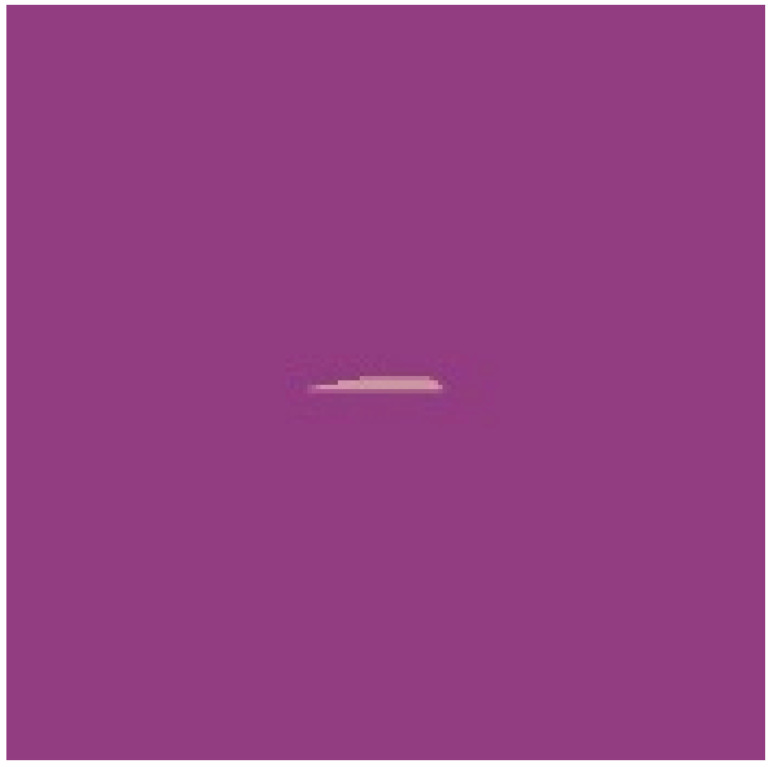	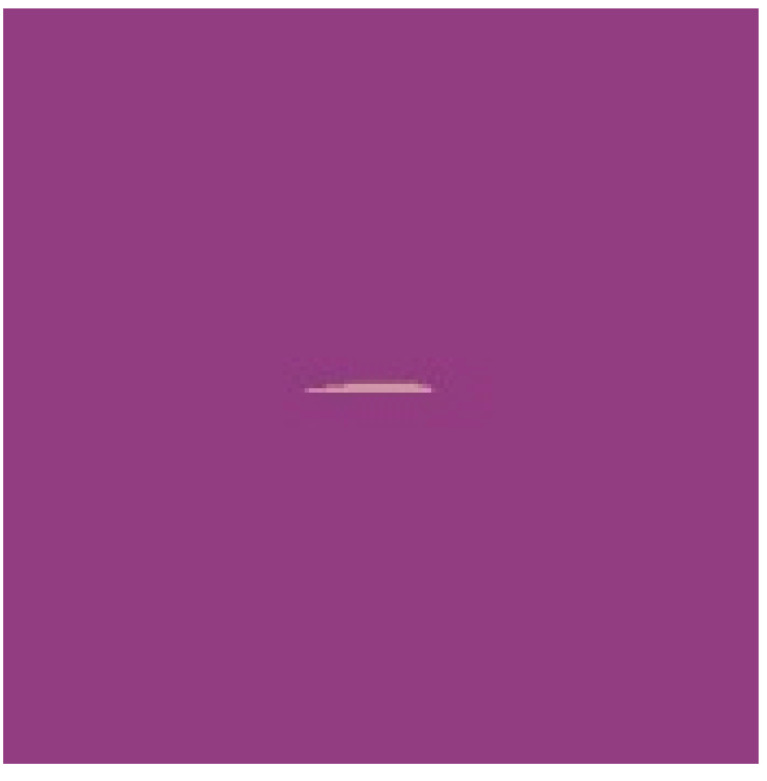	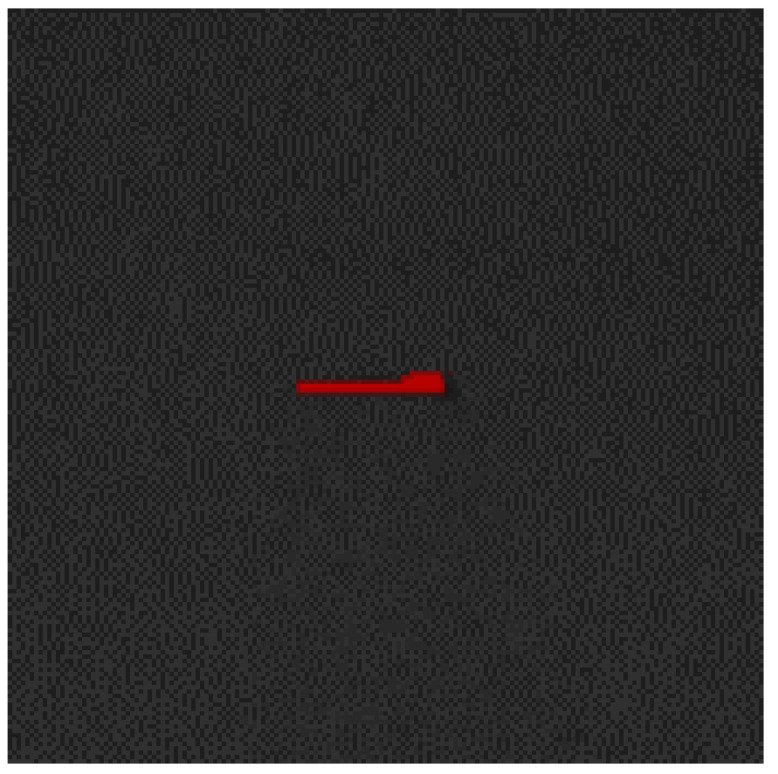	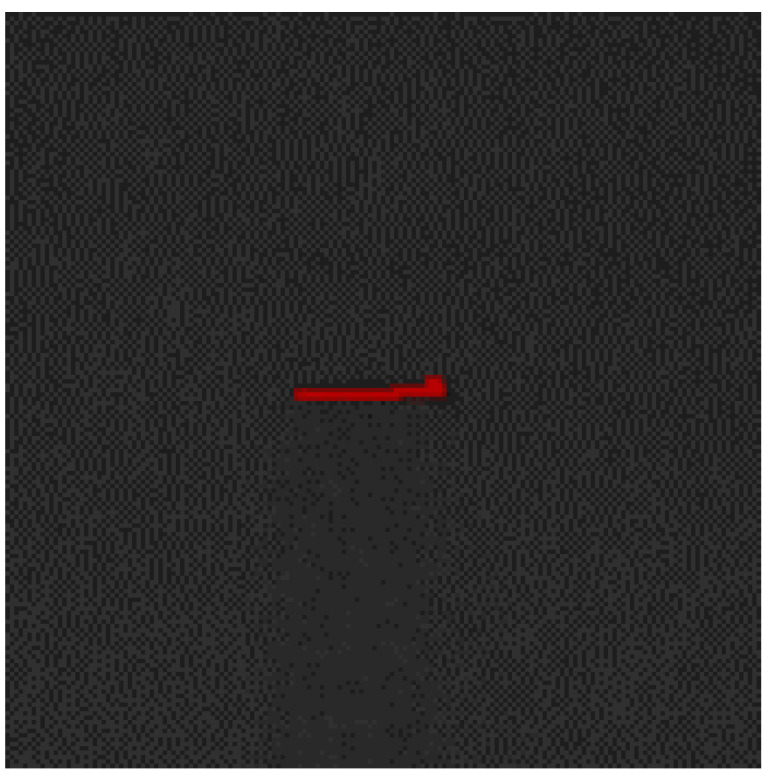	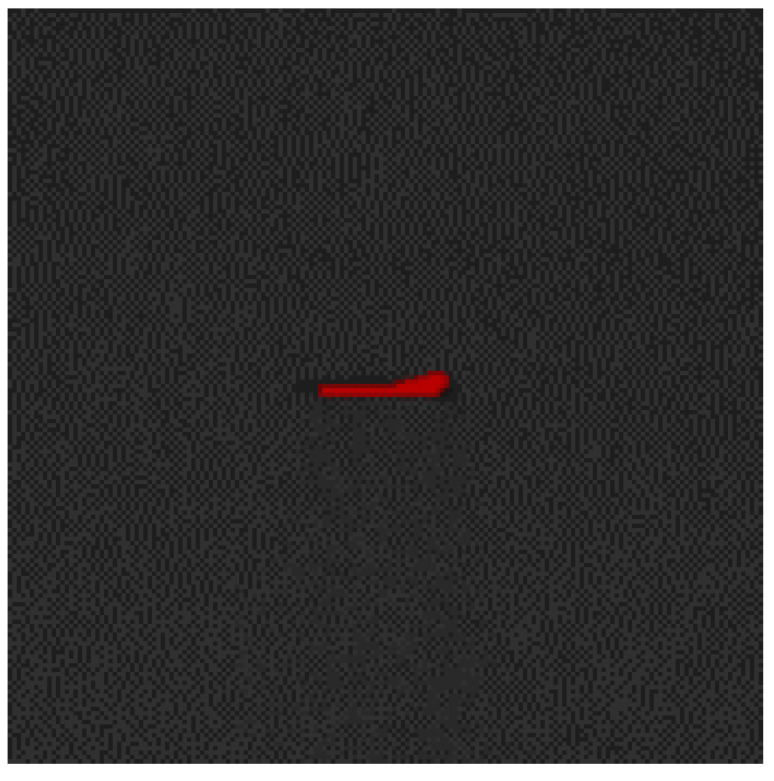
Soft	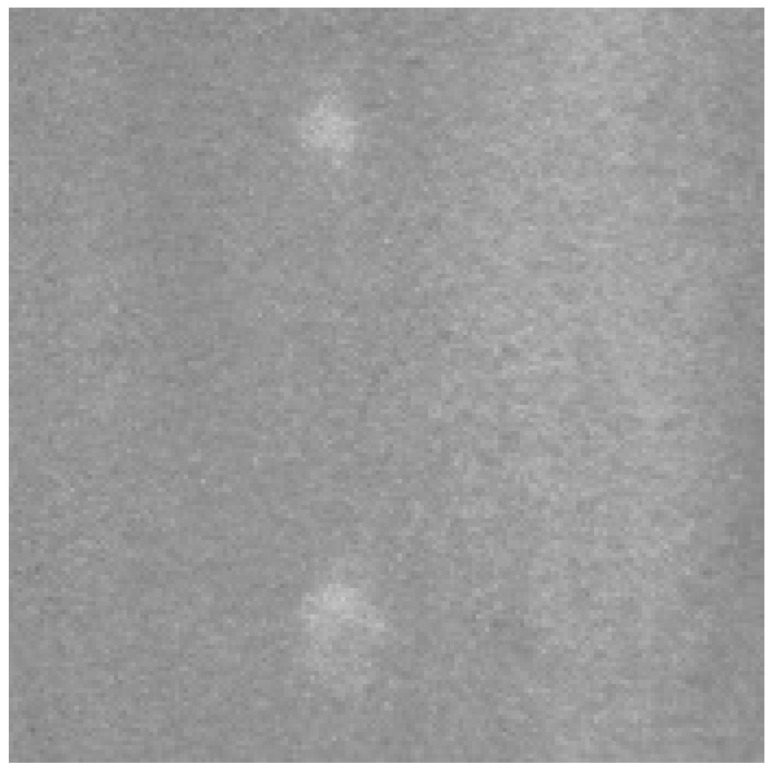	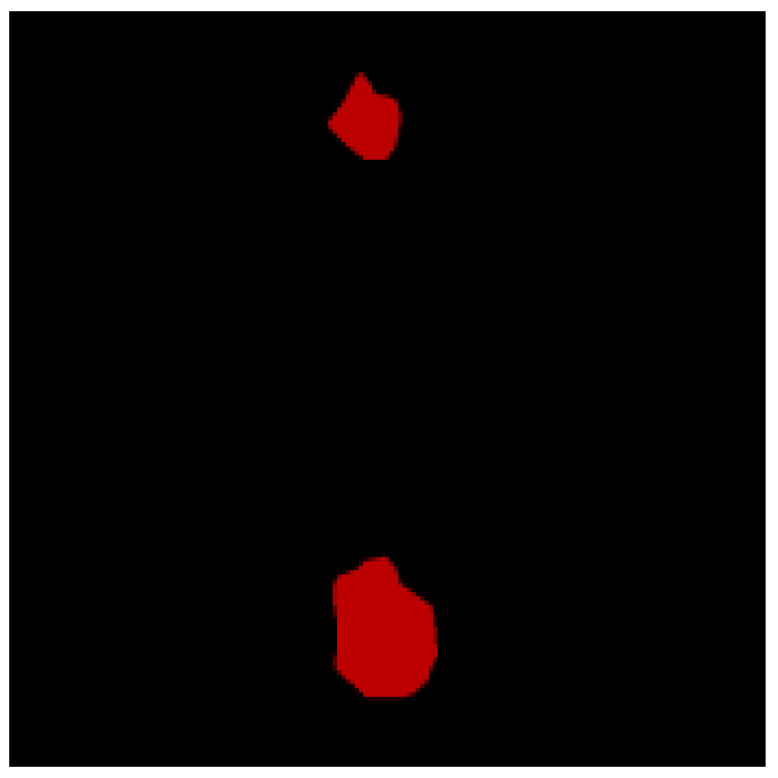	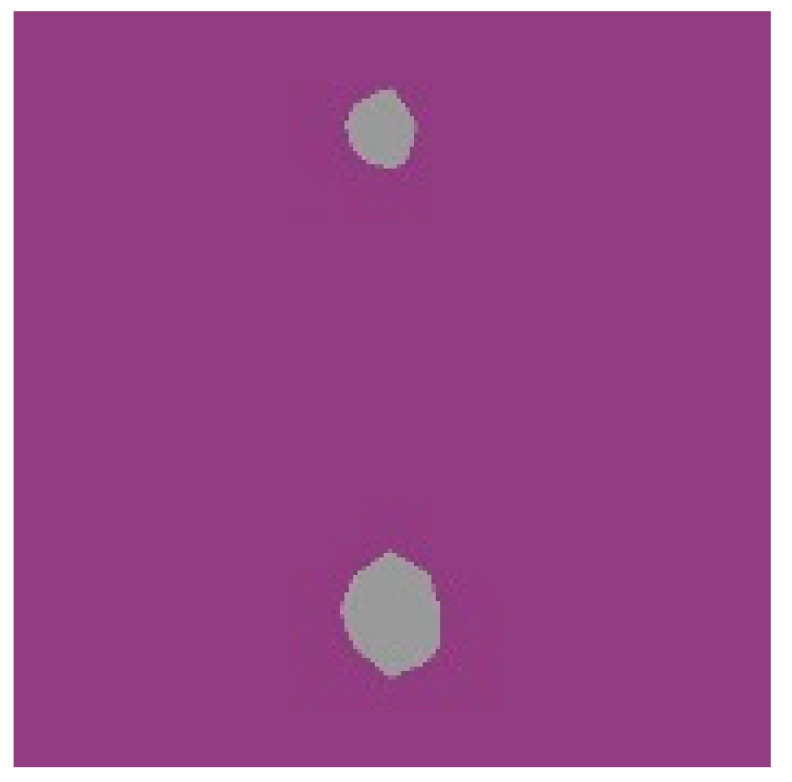	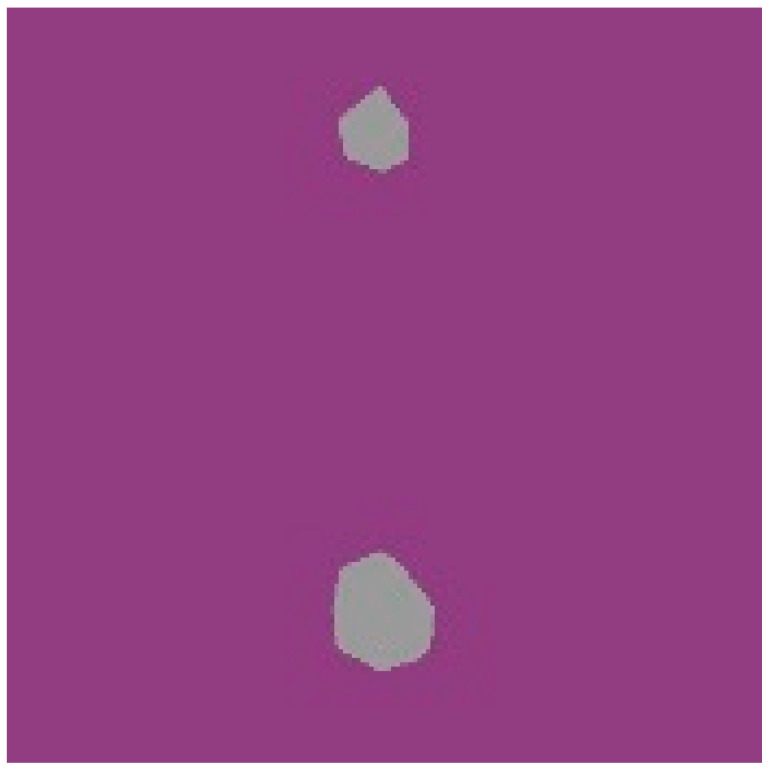	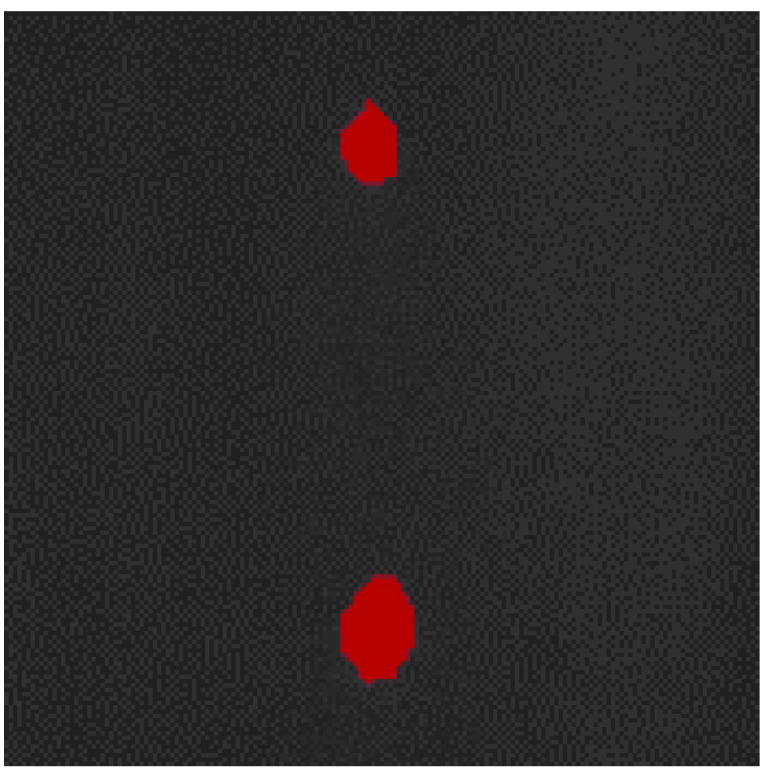	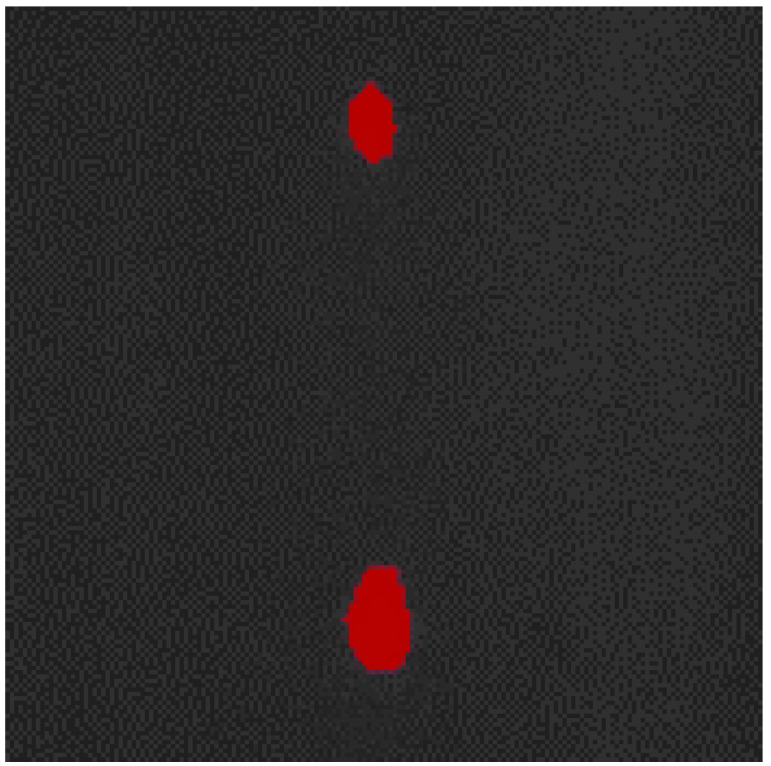	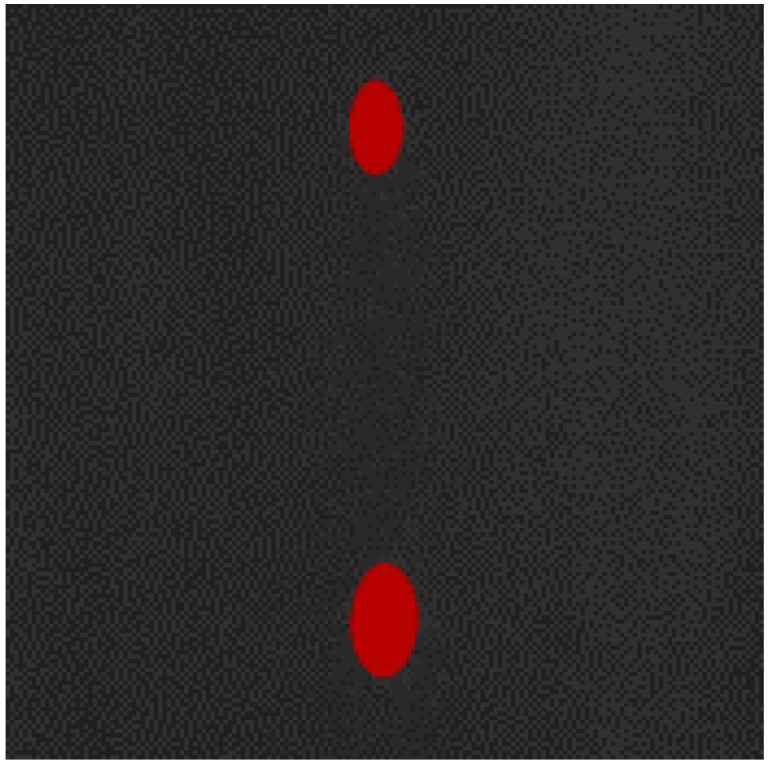

**Table 7:** Detection rate of surface defects of five classes of particleboard.

	Defective Board					Average Value
Defect Classes	SandLeakage	BigShavings	GlueSpot	OilPollution	Soft	
Detection rate	96%	97%	100%	98%	100%	98.2%
Missed detection rate	4%	3%	0	2%	0	1.8%

## Data Availability

Not applicable.
